# Injectable antibacterial conductive nanocomposite cryogels with rapid shape recovery for noncompressible hemorrhage and wound healing

**DOI:** 10.1038/s41467-018-04998-9

**Published:** 2018-07-17

**Authors:** Xin Zhao, Baolin Guo, Hao Wu, Yongping Liang, Peter X. Ma

**Affiliations:** 1 Frontier Institute of Science and Technology and State Key Laboratory for Mechanical Behavior of Materials, Xi’an Jiaotong University, 710049 Xi’an, China; 2 Department of Orthopaedics, the First Affiliated Hospital, College of Medicine, Xi’an Jiaotong University, 710061 Xi’an, China; 3 Department of Biomedical Engineering, University of Michigan, Ann Arbor, MI 48109 USA; 4 Department of Biologic and Materials Sciences, University of Michigan, 1011, North University Ave., Room 2209, Ann Arbor, MI 48109 USA; 5 Macromolecular Science and Engineering Center, University of Michigan, Ann Arbor, MI48109 USA

## Abstract

Developing injectable antibacterial and conductive shape memory hemostatic with high blood absorption and fast recovery for irregularly shaped and noncompressible hemorrhage remains a challenge. Here we report injectable antibacterial conductive cryogels based on carbon nanotube (CNT) and glycidyl methacrylate functionalized quaternized chitosan for lethal noncompressible hemorrhage hemostasis and wound healing. These cryogels present robust mechanical strength, rapid blood-triggered shape recovery and absorption speed, and high blood uptake capacity. Moreover, cryogels show better blood-clotting ability, higher blood cell and platelet adhesion and activation than gelatin sponge and gauze. Cryogel with 4 mg/mL CNT (QCSG/CNT4) shows better hemostatic capability than gauze and gelatin hemostatic sponge in mouse-liver injury model and mouse-tail amputation model, and better wound healing performance than Tegaderm™ film. Importantly, QCSG/CNT4 presents excellent hemostatic performance in rabbit liver defect lethal noncompressible hemorrhage model and even better hemostatic ability than Combat Gauze in standardized circular liver bleeding model.

## Abstract (web summary)

To improve trauma survival and surgical outcomes, hemostatic agents are needed. Here, the authors report on the development of injectable, biocompatible carbon nanotube reinforced quaternized chitosan cryogels with shape memory, conductivity and antibacterial properties for hemostatic control.

## Introduction

Hemorrhage control is a significant concern of military and civilian trauma centers across the world^1^, and uncontrolled hemorrhage leads to over 30% of trauma deaths world-wide and more than half of those occur before emergency care can be reached^2,3^. Thus, employing hemostatic agents to rapidly and effectively control the hemorrhage is very important for trauma emergency. An ideal hemostatic agent should not only quickly control massive hemorrhage from large arteries, veins, and visceral organs but also should be biocompatible, ready and easy to use, lightweight, stable, and inexpensive^1^. Although the current hemostatic agents including cyanoacrylates, glutaraldehyde cross-linked albumin^2^, zeolite-based QuickClot^3^, fibrin based bandages or gelatin-based hemostatic agents^4,5^ have been proven to be highly effective in stopping the hemorrhage, they are often ineffective for deep wounds incurred by small-caliber firearms, improvised explosive devices in battlefield and everyday life^6^, which are irregularly shaped and noncompressible^7^.

To address these issues, new hemostatic technologies were developed. XStat™ device, an applicator filled with numerous compressed cellulose sponges, can rapidly expand to fill and apply pressure to deep, noncompressible wounds^8^. Also, many other shape memory polymer foams as wound dressings or hemostatic agents were developed and presented good hemostatic capability^9–11^. However, XStat™ consists of miniature sponges, and need much more time to remove each individual sponge from the wound bed^7^. Besides, shape memory polymer foams often show inherently limited capacity for absorbing fluid^7^, and they need to take decades of seconds to recover their shapes^7,9–11^, which may prolong the hemostatic time and lose more blood. Thus, development of a user-friendly shape memory hemostatic material with instantaneous and high blood absorption capacity and fast shape recovery capacity to rapidly cease the noncompressible hemorrhage are still highly needed.

Cryogels possess inherent interconnected macroporous structure, and the characteristic structure allows water freely flow in and out of the cryogel, by which way the cryogel shape can be fixed by squeezing out of the free water and fast recovery to its original shape by absorbing water^12–14^. Thus, cryogel presents remarkable potential as shape memory hemostatic agent. However, although a lot of cryogels have been developed for biomedical applications^12,15–19^, there is no report about using cryogels for hemostatic application. Thus, cryogel hemostatic dressings developed by cheap materials with high inherent hemostatic ability are highly anticipated, while it still remains a challenge.

The cryogels often present relative weak mechanical strength for their macroporous morphology produced by formation of ice crystals in cryotropic gelation^12,13,18,19^. Nanocomposite hydrogels and cryogels were recently reported by using carbon nanotube (CNT) as the additive to enhance the mechanical property of the materials^13,20–23^. Moreover, the introduction of CNT can also endow the cryogel with excellent conductivity^24^ and NIR stimuli-responsive ability. On the other hand, the growing incidence of infection by antibiotic-resistant bacteria strains, is another challenge facing caregivers in combat trauma wounds^4,25,26^. Hemostatic agents with inherent antibacterial ability will show better performance than broad spectrum antibiotics in wound anti-infection under combat conditions^4^. Quaternized chitosan (QCS) exhibits good water solubility and enhanced antibacterial activity than chitosan^27,28^, and it performs good hemostatic effect and biocompatibility in vivo^29,30^. These properties of QCS suggest that it is an excellent candidate to prepare antibacterial cryogel hemostatic dressing, which has not been reported. Thus, developing multifunctional CNT-reinforced QCS based shape memory cryogel hemostatic dressing to stop the hemorrhage of deep noncompressible wounds is highly desirable in this field.

In this study, we aim to develop injectable antibacterial and conductive nanocomposite cryogels based on CNT-reinforced glycidyl methacrylate (GMA) functionalized quaternized chitosan (QCSG) with robust mechanical property, rapid blood-triggered shape recovery, rapid blood absorption speed, excellent NIR stimuli-responsivity, and we further demonstrate their great potential for in vivo lethal noncompressible hemorrhage hemostasis and wound healing applications. The cryogels show much better blood-clotting ability, blood cell and platelet adhesion and activation, than gauze and commercial gelatin hemostatic sponge in vitro. Furthermore, the hemostatic time and blood loss are evaluated in vivo to investigate the hemostatic effects of the cryogels in mouse liver injury model, mouse-tail amputation model, New Zealand rabbit liver defect lethal noncompressible hemorrhage model, and standardized circular New Zealand rabbit liver bleeding model, while the wound closure and histopathological examinations are evaluated in vivo to investigate the therapeutic effects of the cryogels in a mouse full-thickness skin defect model. All these results indicate the great potential of these cryogel as noncompressible hemorrhage hemostasis dressing and wound healing application.

## Results

### Synthesis of antibacterial conductive nanocomposite cryogel

**Fig. 1:**
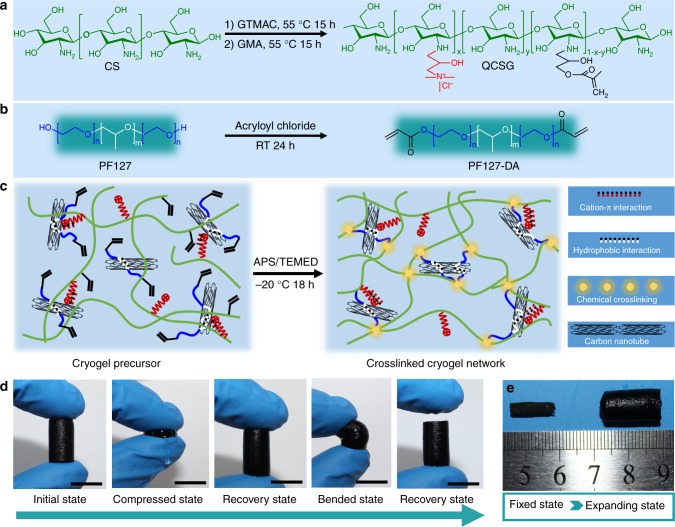
Schematic representation of QCSG/CNT cryogel synthesis. **a** Synthesis of QCSG copolymer. GTMAC and GMA with a fixed 0.5:1 molar ratio of GMA to amino groups and varying the GTMAC: amino groups from 1:1 (coded as QCSG1) to 2:1 (coded as QCSG2) and 3:1 (coded as QCSG3). **b** Synthesis of PF127-DA copolymer. **c** Preparation of QCSG/CNT cryogel. **d** Photographs of the compression and bending resistance capability of QCSG/CNT4 cryogels: initial state, compressed state by squeezing out of the free water, recovery state by absorbing water, bending and squeezing out of part free water, and recovery state after absorbing water. **e** Shape-fixed state after removing the free water (left) and expanding state after absorbing water (right). Scale bar: 1 cm

We prepared a series of CNT-reinforced antibacterial and conductive nanocomposite cryogels with rapid blood-triggered shape recovery and excellent NIR stimuli-responsivity as injectable shape memory hemostatic dressings based on QCSG and CNT (Fig. 1). Firstly, three QCSG copolymers were synthesized via one-pot reaction of chitosan (Fig. 1a, and Supplementary Table 1). QCSG3 with the quaternary amination degree of 46% showed excellent antibacterial activity (minimum inhibitory concentrations (MICs) of 20 µg/mL for *S. aureus* and 40 µg/mL for *E. coli*)^27,30^ and water solubility, and it was chosen to prepare the antibacterial cryogel in the further study (Supplementary Table 1). Secondly, CNT was well dispersed under ultrasound with the assistance of the diacrylate capped PF127 (PF127-DA) in water (Supplementary Fig. 1, and Supplementary Fig. 2). Thirdly, QCSG solution was fine mixed with PF127-DA/CNT dispersion liquid. The QCSG and PF127-DA/CNT dispersion was then placed at −20 ^°^C for cryopolymerization after adding APS/TEMED to form cryogel (Fig. 1c). QCSG served as the basic network of hemostatic cryogel, and QCSG with abundant positively charged quaternary ammonium groups would endow the cryogel with inherent antibacterial activity and excellent hemostatic capability^3,27,30–33^. Moreover, the CNT in the cryogel could provide the cryogel with hydrophobic drug encapsulation ability, NIR stimuli-responsivity, as well as good mechanical strength (Fig. 1d). Furthermore, due to the cryogel’s interconnected and macroporous structure, the cryogel could show rapid water-triggered shape memory property and high water absorption ability (Fig. 1e), as well as injectability (Supplementary Movie 1, Supplementary Movie 2, Supplementary Movie 3, Supplementary Movie 4 and Supplementary Fig. 3). Four cryogels with varying the CNT concentrations were synthesized (Supplementary Table 2). Chemical structure of the cryogels was confirmed by FTIR (Supplementary Fig. 4).

### Swelling ratio and mechanical properties of cryogels

**Fig. 2:**
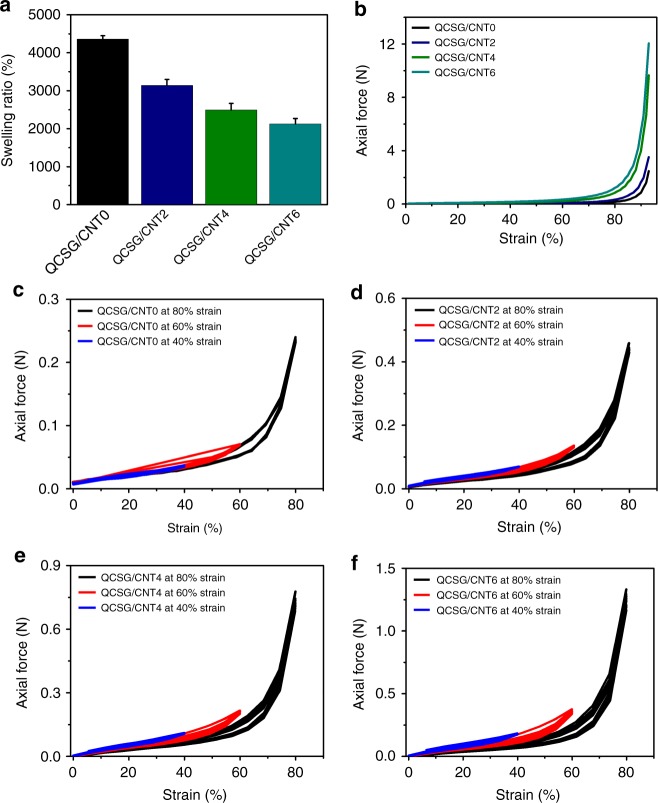
Swelling and mechanical property of the cryogels. **a** Swelling ratios of the cryogels. **b** The uniaxial compression stress–strain curves of the cryogels. QCSG/CNT0 presented the lowest axial force of 2.5 N when bearing a 93% compression strain, while the axial forces significantly increased from 3.5 to 9.6 and 12.0 N with the increase of CNT content from 2 mg/mL to 6 mg/mL in the cryogel networks. Furthermore, all of the four cryogels kept stable and intact after the test. The stress–strain cycling curves of QCSG/CNT0 (**c**), QCSG/CNT2 (**d**), QCSG/CNT4 (**e**), and QCSG/CNT6 (**f**) with three different compression strains of 40%, 60%, and 80%, respectively. Error bar indicates s.d. (*n* = 3)

Cryogels with interconnected and macroporous structure can present fast water uptake capability^13,19,34^. Thus the shape-fixed cryogel can be used to rapidly absorb the wound exudate to reduce the bacterial infection and promote healing^7,35^. Especially, the cryogel’s rapid water absorption ability was also hypothesized to concentrate clotting factors within the cryogel thus to enhance the rate of hemostasis^7,36^. The water absorption ability of the prepared cryogels was shown in Fig. 2a. QCSG/CNT0 had the highest swelling ratio of 4400%, and the swelling ratios of the QCSG/CNT cryogels gradually deceased from 3100 to 2500 and 2100% with the increase of PF127-DA/CNT content in the cryogels. This was due to the gradually increased crosslinking density and increased dry weight in per unit volume of the swollen cryogels when increasing PF127-DA/CNT content in the cryogel precursor.

Cryogels prepared by pure natural product generally presented weak mechanical strength, which might hinder their application for hemostasis and wound healing. However, by incorporating CNT into the cryogel network can significantly reinforce the mechanical properties of the cryogels^13^. The cryogels’ compression stress–strain test was performed to evaluate the mechanical strength of the cryogels. These results in Fig. 2b demonstrated the significant reinforcement of QCSG/CNT cryogel by CNT and good mechanical stability of the cryogels. Besides, in order to further evaluate the high resilience with fast recovery and robustness of the interconnected macroporous cryogels, the dynamic stress–strain behavior of the cryogels was performed for 10 cycles by applying three different strains of 40%, 60%, and 80%, respectively. As shown in Fig. 2c–f, all the four cryogels showed no obvious recovery loss when applying 40% and 60% strains, and all the cryogels still presented good shape and elasticity, suggesting their good compression resilience under small compression strains. When increasing the compression strain from 60% to 80%, all the four cryogels presented increasing recovery loss from 3.3 to 6.2, 11.8, and 12.4% with the increase of CNT in the cryogels. Moreover, their great robustness was confirmed by 100 cycles test (Supplementary Fig. 5) and were also presented in Supplementary Movie 5 and Supplementary Movie 6. The rheological properties of the cryogels in Supplementary Fig. 6 also suggested their stable cryogel networks. The CNT was stably trapped in the cryogel matrix when bearing continuous and dynamic compression (Supplementary Note 1). Furthermore, the cryogel as hemostatic agents will not cause severe pressure and additional injury to soft tissue during the application (Supplementary Note 2).

The mechanism of the high resilience and rapid recovery behavior was because of the elastic macroporous and soft-hard bicontinuous network structure of QCSG/CNT cryogel^13^ (Supplementary Note 3). Thus, all these results demonstrated that the CNT-reinforced cryogels possessed good mechanical strength (high resilience and rapid recovery) and stability, revealing their potential applications as injectable blood-triggered shape recovery hemostatic agent in deep wound hemostasis.

### Conductivity and related photothermal property of cryogels

**Fig. 3:**
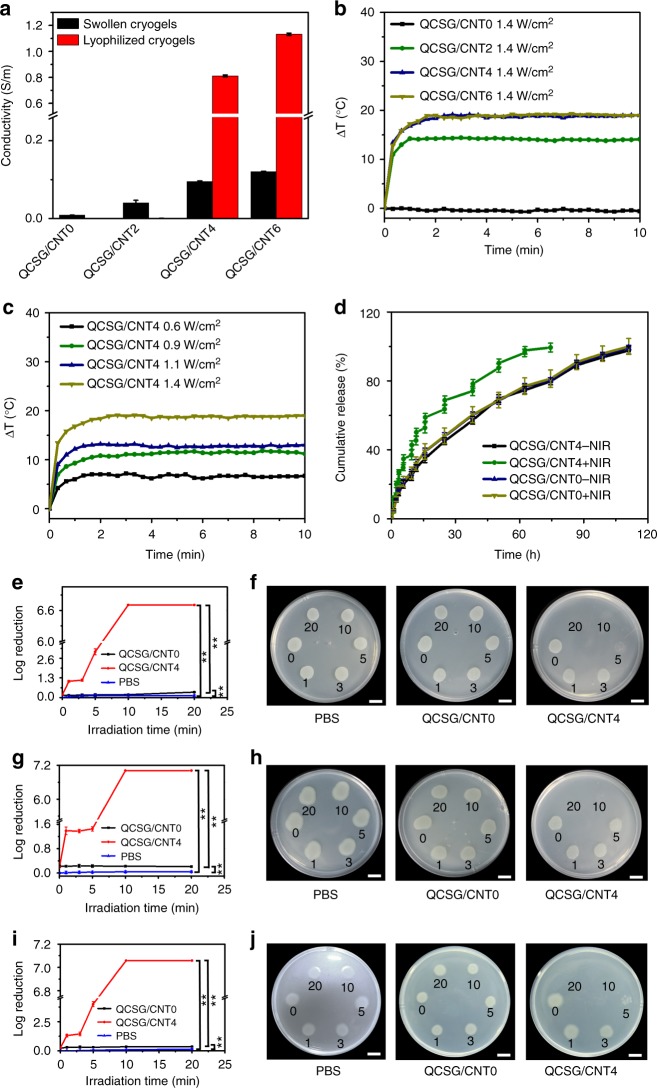
Conductivity, photothermal property, release behavior, and antibacterial activity of the cryogels. **a** Conductivity of the cryogels at wet state and dry state. **b** ΔT-NIR irradiation time curves of the cryogels using a constant light intensity of 1.4 W/cm^2^. **c** ΔT-NIR irradiation time curves of QCSG/CNT4 varying the light intensity from 0.6 to 0.9, 1.1 and 1.4 W/cm^2^, respectively. **d** Spontaneous release profiles and NIR-triggered release profiles of ibuprofen from QCSG/CNT0 and QCSG/CNT4. Both QCSG/CNT0 with and without NIR irradiation and QCSG/CNT4 without NIR irradiation  showed similar sustained release profiles as long as 111 h in PBS. However, QCSG/CNT4 presented obvious burst release after applying 10 min NIR irradiation at each time point and completely released the drug within 74 h. When stopping the irradiation, ibuprofen’s release profile returned to its common slow pattern. The killing-time curves of **e** *S. aureus*, **g** *E. coli*, and **i** *P. aeruginosa* for the cryogel groups and PBS group after exposed to NIR irradiation (1.4 W/cm^2^) for 0 min, 1 min, 3 min, 5 min, 10 min, and 20 min, respectively. Photographs of the survival **f** *S. aureus*, **h** *E. coli*, and **j** *P. aeruginosa* for the cryogel groups and PBS group after exposed to NIR irradiation (1.4 W/cm^2^) for 0 min, 1 min, 3 min, 5 min, 10 min, and 20 min, respectively. Scale bar: 1 cm. **P < 0.01 using Student's *t*-test (two-sided). Error bar indicates s.d. (*n* = 3)

CNTs possess remarkable conductivity and NIR-responsive photothermal property^37^. Our previous reports have demonstrated the positive effect of conductive materials^38–42^ on tissue engineering including wound healing applications^43^. The wet QCSG/CNT0 cryogel presented the lowest conductivity of 8.5 × 10^−3^ S/m from amino groups and quaternary ammonium groups of QCSG (Fig. 3a). When introducing CNT and varying CNT content from 2 to 4 and 6 mg/mL, the conductivity of the cryogels gradually increased from 4.0 × 10^−2^ to 9.5 × 10^−2^ and 1.2 × 10^−1^ S/m, revealing the obvious contribution of CNT to the cryogels’ conductivity. When drying the cryogels, all the cryogels’ ionic conductivity from QCSG disappeared. Thus, the conductivity of QCSG/CNT0 dramatically decreased (2.1 × 10^−7^ S/m), and QCSG/CNT2 showed decreased conductivity of 5.0 × 10^−4^ S/m due to its lowest CNT content. However, QCSG/CNT4 and QCSG/CNT6 presented significantly higher conductivity of 8.1 × 10^−1^ S/m and 1.1 S/m than their wet states, respectively, which might be explained for the higher connective network among CNT. The conductivity of QCSG/CNT4 and QCSG/CNT6 would be effective to transmit electrical signals in wound tissue, which will promote wound healing process^43^. The QCSG/CNT4 presented a conductivity of 0.19 S/m at 100 Hz, while QCSG/CNT0 showed a very-low conductivity of 3.4 × 10^−7^ S/m from impedance curves in Supplementary Fig. 7. The results are in agreement with those from the digital 4-probe tester measurements.

The photothermal capacity of the cryogels was evaluated. The ΔT-NIR irradiation time curves of the cryogels were shown in Fig. 3b. QCSG/CNT0 showed no temperature increase after 10 min NIR irradiation (1.4 W/cm^2^), while with the increase of CNT content from 2 to 4 and 6 mg/mL, the equilibrium ΔTs increased from 14 °C to 19 °C, demonstrating the excellent photothermal efficiency of CNT-reinforced cryogels. Besides, when QCSG/CNT4 was irradiated by NIR varying the light intensity from 0.6 to 0.9, 1.1, and 1.4 W/cm^2^, the ΔTs gradually increased from 7 to 11, 13, and 19 °C, revealing the adjusted photothermal effect of CNT-reinforced cryogels (Fig. 3c). Furthermore, the heat maps of the cryogels after 10 min NIR irradiation in Supplementary Fig. 8 were also consistent with the results in Fig. 3b, c, and a core central region with maximum temperatures from 32–44 °C (corresponding ΔT from 7–19 °C) was surrounded by zones with a large temperature gradient from the heat maps, which favors localized heat treatment^44^.

Above 50 °C, the enzymes in bacteria become denatured and their proteins and lipids on the cell membranes will be damaged, eventually causing bacterial death^44,45^. The NIR-assisted photothermal antibacterial activity of the QCSG/CNT4 (ΔT of 19 °C) was evaluated with QCSG/CNT0 and PBS as control groups as shown in Fig. 3e–j (Supplementary Note 4). When introducing CNT as photothermal contrast agent, QCSG/CNT4 showed significantly increased bacteria log reductions from 0.17 to 1.07 (92% killing ratio) for *S. aureus*, from 0.20 to 1.39 (96% killing ratio) for *E. coli*, and from 0.25 to 1.31 (95% killing ratio) for *P. aeruginosa*, respectively, after only 1 min NIR irradiation. Further increasing the irradiation time to 10 min, QCSG/CNT4 groups showed 100% bacteria killing ratios for all the three bacteria and presented log reductions of 6.71, 7.01, and 7.06 for *S. aureus*, *E. coli* and *P. aeruginosa*, respectively. Photographs of the survival bacteria in Fig. 3 showed the similar results. The mechanism of the NIR-assisted antibacterial activity was that CNT can absorb NIR irradiation and efficiently converted it into localized heat to photothermally lyse the bacteria^44^. Furthermore, the short irradiation time between 1 min and 10 min according to the wound infection degree will present negligible damage on human body but high-efficiency on antimicrobial infection. These results demonstrated that the CNT-reinforced cryogel possessed excellent NIR photothermal capacity, which greatly enhanced antibacterial activity for both gram-positive bacteria and gram-negative bacteria even when bearing a challenge of 10^8^ CFU/mL bacteria.

Furthermore, ibuprofen as a widely used non-steroidal anti-inflammatory analgesic in wound^46–49^ was chosen to endow the cryogel hemostatic agents with analgesic effect, and the release behavior of ibuprofen from the cryogels with and without NIR irradiation (1.4 W/cm^2^) was studied (Fig. 3d). These release data demonstrated the sustained release behavior of analgesic from CNT-reinforced cyrogel, and analgesic burst release on demand by NIR stimulus (Fig. 3d). Thus the CNT-reinforced cryogel could be used as potential carrier for sustained release and NIR-triggered on demand release of analgesic for relieving wound pain during hemostasis and wound healing applications.

### Water- and blood-triggered shape memory of cryogels

**Fig. 4:**
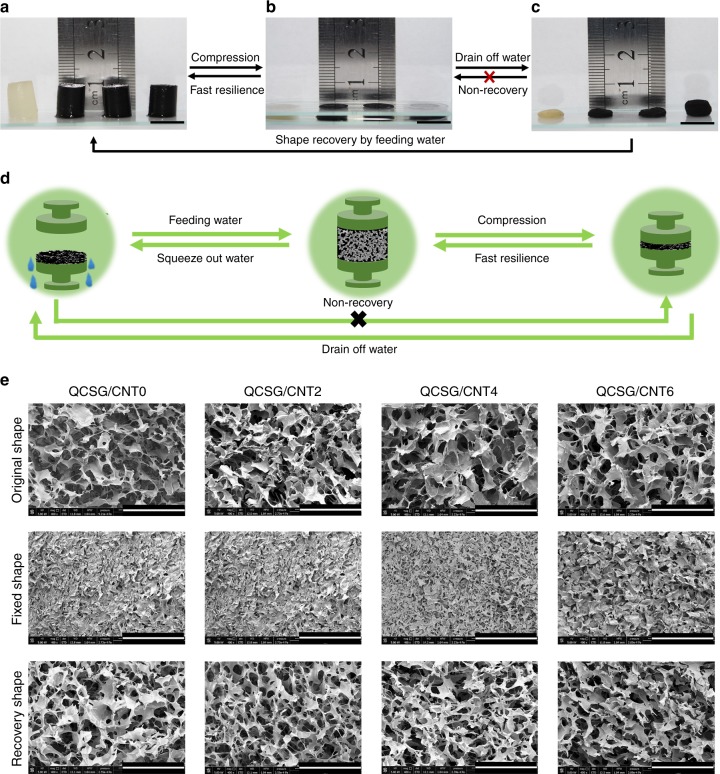
Shape memory properties of the cryogels. **a–c** Fast resilience and macroscopical shape memory property of the cryogels. Scale bar: 1 cm. **d** Schematic representation of the shape memory mechanism of the cryogel. **e** Microtopography of the cryogels in original state, shape-fixed state and shape recovery state after fixing. Scale bar: 400 μm. All the four cryogels under free shape showed interconnected macroporous structure with similar pore size between 100–200 µm. Compared to their shapes under free situation, all the shape-fixed cryogels presented collapsed and almost closed pores except QCSG/CNT6 still remained unclosed pores with reduced pore size. However, all the four shape-fixed cryogels still kept unbroken network. After absorbing water, all the cryogels’ morphologies were similar to those in original state

Shape memory hemostatic agents can present unique property in hemostasis application due to their capacity to be delivered to the wound site in the shape-fixed state by an injector and recover their shape to the expanded geometry upon contacting with bleeding blood in human body^7,9–11^. Especially, the volume expansion of the device over short period of time would allow for easy delivery into narrow, penetrating wounds, and subsequent expansion to completely fill abnormal wound boundaries^7,9–11^. Considering the excellent water-triggered shape memory property of cryogels^7,35,50^, we evaluated the shape memory property of the as-prepared cryogels qualitatively and quantitatively including the volumetric expansion ratio, shape fixity ratio, recovery ratio, and recovery time. As shown in Fig. 4, the shape-fixed state of the four cryogels could be conveniently achieved by simply compressing and absorbing the water squeezed out from the cryogels (Fig. 4b, c). Then, they immediately recovered to their original state less than 1 s by reabsorbing water (Fig. 4a and Supplementary Movie 7). Similar to the water-triggered shape recovery, QCSG/CNT0 and QCSG/CNT4 also showed rapid shape recovery after contacting and absorbing blood (Supplementary Movie 8, Supplementary Movie 9 and Supplementary Fig. 9). These results demonstrated the excellent shape memory property and rapid recovery speed of the cryogels. Besides, the quantitatively shape memory results were shown in Supplementary Table 3. All the shape-fixed cryogels could rapidly recover their original shapes (100% recovery ratio) less than 1 s (Supplementary Movie 7). Furthermore, the shape-fixed QCSG/CNT0 and QCSG/CNT4 (with a free shape diameter of 5 mm) could be injected using an injector with diameter of about 1.5 mm. After injection, the cryogels still maintained their original shapes and kept stable (Supplementary Fig. 3, Supplementary Movie 3, and Supplementary Movie 4), revealing their great injectability.

The microtopography recovery of the cryogels at their original shapes, fixed shapes and recovered shapes after fixation were further confirmed in Fig. 4e. The shape memory mechanism of the cryogel was schematically represented in Fig. 4d. The shape memory capability was attributed to the reversible collapse of the pores within the cryogel matrix, in which the macroporous sponge-like structure allowed water flow out/in freely, possessed high polarity to absorb water and good resilience for shape recovery^13,14^. The processes of compressing caused efflux of water from interconnected pores and collapse of the cryogel structure. After removing the compressive stress and absorbing the water, the stored elastic energy is released and the cryogel presents shape-fixed state. Then, when the shape-fixed cryogel contacts water, the water will be immediately reabsorbed into the cryogel’s matrix to recover the cryogel’s shape and structure. All these above results demonstrated that the cryogels possessed excellent water -triggered shape memory capability, especially for QCSG/CNT0, QCSG/CNT2, and QCSG/CNT4 simultaneously possessing excellent volumetric expansion ratio, shape fixity ratio, recovery ratio, and recovery time. Thus, QCSG/CNT0, QCSG/CNT2, and QCSG/CNT4 showed huge potential as cryogel hemostatic agent with rapid blood-triggered shape recovery for hemostasis applications.

### Hemocompatibility and cytocompatibility of the cryogels

**Fig. 5:**
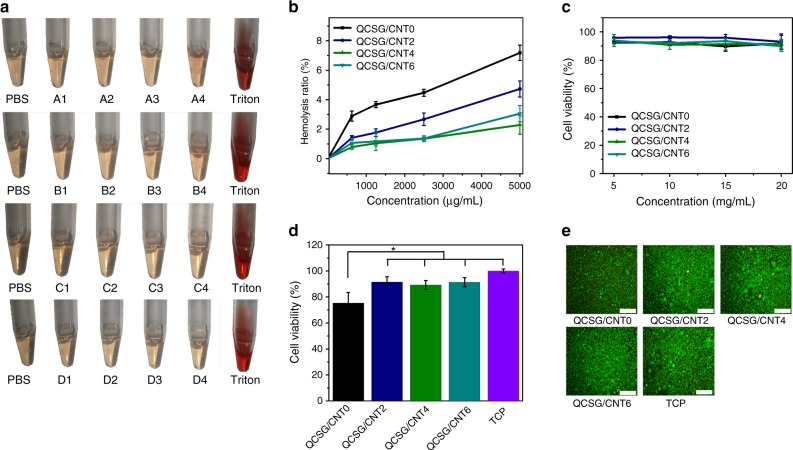
Biological properties assays for the cryogels. **a** Photographs from hemolytic activity assay of the cryogels using PBS as negative control and Triton X-100 as positive control. A: QCSG/CNT0, B: QCSG/CNT2, C: QCSG/CNT4, and D: QCSG/CNT6, and the number after the letter stands for the cryogel dispersion liquid concentration that 1 represents 625 µg/mL, 2 represents 1250 µg/mL, 3 represents 2500 µg/mL, and 4 represents 5000 µg/mL, respectively. **b** Hemolytic percentage of the cryogels’ dispersion liquids at different concentrations. When the cryogel dispersion concentrations were equal to or less than 1250 µg/mL, all the three CNT-contained cryogels just presented less than 1.8% hemolysis, which was lower than that of QCSG/CNT0 (3.6% hemolysis). When increasing the dispersion concentration to as high as 5000 µg/mL, the three CNT-contained cryogels just presented no more than 4.8% hemolysis. However, the hemolysis ratio of QCSG/CNT0 reached to 7.2%. **c** Cytocompatibility evaluation of the cryogels’ extracts for L929 cells. When changing the cryogel extracts’ concentrations from 5 to 10, 15, and 20 mg/mL, all the four cryogels presented more than 90% L929 cell viability compared with TCP control group (*P* > 0.05). **d** Cytocompatibility evaluation of the cryogels when contacted with the cryogel disks. **e** LIVE/DEAD staining of L929 cells after contacted with the cryogels for 24 h. Scale bar: 200 µm. *P < 0.05 using Student's *t*-test (two-sided). Error bar indicates s.d. (*n* = 4)

In vitro hemolysis assay is a universal method to evaluate the hemocompatibility of materials^51^. The hemolysis ratios of the four cryogels’ dispersion liquids with the concentrations varying from 625 to 1250, 2500, and 5000 µg/mL were tested. The macroscopical color of centrifugally obtained supernatants for all the cryogel groups, negative PBS group and positive Triton X-100 group was shown in Fig. 5a. All the four cryogel groups presented light yellow similar to PBS control group, while the positive group was bright red. For the quantitative data as shown in Fig. 5b, QCSG/CNT4 showed the lowest hemolysis ratio of 2.3% when bearing a 5000 µg/mL dispersion concentration, revealing its best hemocompatibility in the four cryogels due to its balanced CNT concentration. Although the cryogels presented different hemolysis ratios, they showed better hemocompatibility than the reported hemostatic materials^52,53^. These hemolysis results demonstrated the excellent hemocompatibility of materials as hemostatic agent and wound dressing.

For the applications of hemostatic agent and wound dressing, the materials should possess good cytocompatibility. Two methods including a leaching pattern and a direct contact test were used to evaluate the cytocompatibility of as-prepared materials^43,54^. As shown in Fig. 5c, all the four cryogels’ extracts as high as 20 mg/mL had no cytotoxic leaching content. Then, the good cryogel’s cytocompatibility was further demonstrated by a direct contact test as shown in Fig. 5d–e (Supplementary Note 5). Overall, the results from the leaching pattern test demonstrated the non-cytotoxicity of all the extracts of cryogels and the results from direct contact test demonstrated that all the CNT-contained cryogels had excellent cytocompatibility allowing their application as potential trauma hemostatic agents or trauma dressings.

### In vitro blood-clotting performance of the cryogels

**Fig. 6:**
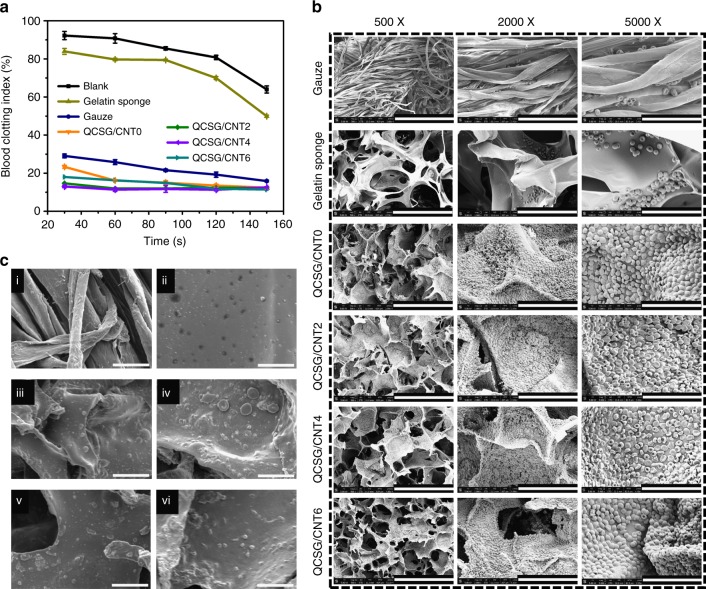
In vitro hemostatic capacity evaluation of the cryogels. **a** In vitro dynamic whole-blood-clotting evaluation of the cryogels and controls. The blank group without any hemostatic agents showed the slowest blood-clotting speed and the highest BCI (blood-clotting index) after 150 s. Compared with blank group, gelatin sponge group showed more than a 14% decrease in BCI after 150 s (*P* < 0.05), while gauze group showed significantly deceased BCI compared to gelatin sponge at each time point (*P* < 0.001). **b** SEM images of hemocyte adhesion on the cryogels and controls. Scale bar: 300 µm for ×500; Scale bar: 100 µm for ×2000; Scale bar: 40 µm for ×5000. **c** SEM images of platelet adhesion on the gauze (ci), gelatin hemostatic sponge (cii), QCSG/CNT0 (ciii), QCSG/CNT2 (civ), QCSG/CNT4 (cv), and QCSG/CNT6 (cvi), respectively. Scale bar: 15 µm. The error bars stand for s.e.m. (*n* = 3)

The blood-clotting capability of the cryogels was evaluated by dynamic whole-blood-clotting test, in which a higher absorbance value of the hemoglobin solution indicates a slower clotting rate^4,52–54^. Gauze as traditional hemostatic agent and commercial gelatin hemostatic sponge were both used as control groups. Interestingly, the QCSG/CNT0 with rapid blood absorption capability showed lower BCI than that of gauze group for each time point (*P* < 0.05) in Fig. 6a, and QCSG/CNT2 and QCSG/CNT4 showed significantly higher blood-clotting capacity than QCSG/CNT0 in first 60 s (*P* < 0.05), and QCSG/CNT6 presented better BCI than QCSG/CNT0 within 30 s (*P* < 0.05). These results demonstrated that QCSG/CNT0 cryogel has effective blood-clotting ability, and introducing CNT further enhanced its blood-clotting ability.

The hemostatic mechanism of the cryogels was further studied by observing the surface adhesion and morphologies of blood cells and platelets on these cryogels, and gauze and gelatin hemostatic sponge were also used as control groups (Fig. 6b, c, Supplementary Note 6)^52,55^. All the four cryogels showed a large number of blood cells adhering to the cryogel surfaces, and the blood cells presented irregularly formed aggregates. For platelet adhesion in Fig. 6c, all the four cryogels showed many platelets adhesion with activated states for their aggregating and changed shape from irregular distinctive disks to circular deformation^52^. With the introduction of CNT into the cryogel QCSG/CNT0, the CNT-contained cryogels showed increased number of platelet adhesion. All the four cryogels with interconnected porous structure and high expansion ratio, can absorb a large amount of blood with rapid blood absorption speed and blood-concentrating effects and enhanced absorption ability for platelets and blood cells, and all these factors contributed to its excellent in vitro blood-clotting effect.

### In vivo hemostatic performance of the cryogels

**Fig. 7:**
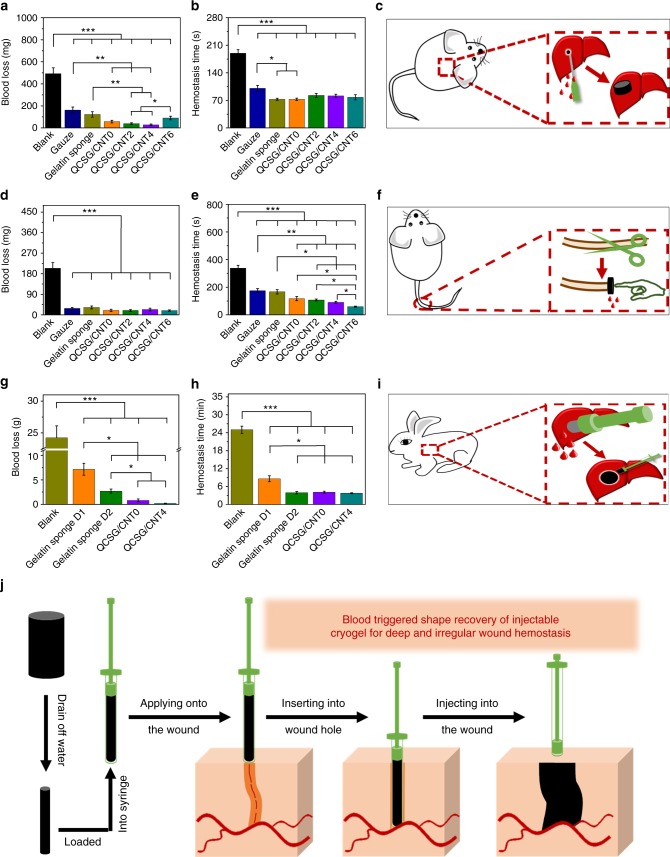
In vivo hemostatic capacity evaluation of the cryogels. Blood loss (**a**) and hemostatic time (**b**) in the mouse liver injury model. The blank group showed the highest blood loss of 492 mg than the other groups (*P* < 0.001). Gauze and gelatin sponge, as two control groups, presented much decreased blood loss of about 163 mg and 123 mg, respectively, when compared to blank group (*P* < 0.001). However, all the four cryogels except for QCSG/CNT6 showed significantly decreased blood loss of 57 mg, 38 mg, and 27 mg than those of gauze and the cryogel QCSG/CNT2 and cryogel QCSG/CNT4 also showed significantly decreased blood loss than that of gelatin sponge (*P* < 0.05). **c** Scheme representation of the mouse liver injury model during hemostasis. Blood loss (**d**) and hemostatic time (**e**) in the mouse-tail amputation model. The blank group (337 s) showed the longest hemostatic time than other groups (*P* < 0.001). All the four cryogels showed shorter hemostatic times than gauze group (176 s) (*P* < 0.01), while all the four cryogels except for QCSG/CNT0 showed shorter hemostatic times than gelatin sponge group (167 s) (*P* < 0.05). **f** Scheme representation of the mouse-tail amputation model during hemostasis; Blood loss (**g**) and hemostatic time (**h**) in the rabbit liver defect lethal noncompressible hemorrhage model. **i** Scheme representation of the rabbit liver defect lethal noncompressible hemorrhage model during hemostasis. **j** Scheme representation of the hemostatic application of injectable shape memory cryogel hemostatic in a deep and irregularly shaped wound model. *P < 0.05, **P < 0.01, ***P < 0.001 using Student's *t*-test (two-sided). The error bars stand for s.e.m. (*n* = 10 for Fig. 7a, b, d, e; *n* = 5 for Fig. 7g, h)

The hemostatic properties of the cryogels were further evaluated by the amount of bleeding and hemostatic time both in the mouse liver injury model and mouse-tail amputation model to obtain the optimized QCSG/CNT cryogel (Fig. 7). For the mouse liver injury model (Fig. 7a–c), among the four cryogels, cryogel QCSG/CNT2 and QCSG/CNT4 presented lower blood loss than that of QCSG/CNT6 (*P* < 0.05). In addition, the hemostatic time of the blank group (190 s) was the longest among all the groups (*P* < 0.001) (Fig. 7b). Gelatin sponge, QCSG/CNT0, QCSG/CNT2, QCSG/CNT4, and QCSG/CNT6 showed hemostatic time of 73 s, 73 s, 83 s, 82 s, and 78 s, and the hemostatic times from gelatin sponge and QCSG/CNT0 were significantly shorter than that of gauze group (101 s) (*P* < 0.05) (Fig. 7b). Besides, there was no significant difference among gelatin sponge, QCSG/CNT0, QCSG/CNT2, QCSG/CNT4, and QCSG/CNT6 (*P* > 0.05). The photographs of the samples after hemostatic applications in Supplementary Fig. 10 were also consistent with the above quantitative data.

For the mouse-tail amputation model mimicking the bleeding situation of ruptured vein, blank group showed the highest blood loss of 204 mg among all the groups (*P* < 0.001) (Fig. 7d–f). However, when applying hemostatic agents including gauze, gelatin sponge, cryogels QCSG/CNT0, QCSG/CNT2, QCSG/CNT4, and QCSG/CNT6, all the six groups showed significantly decreased blood loss varying from 28 to 32, 20, 19, 23, and 19 mg, respectively, compared to blank group (*P* < 0.001) (Fig. 7d). Figure 7e showed the hemostatic times in the mouse-tail amputation model. The cryogels QCSG/CNT0, QCSG/CNT2, QCSG/CNT4, and QCSG/CNT6 presented hemostatic time varying from 117 to 107, 91 and 60 s, respectively. Moreover, QCSG/CNT6 presented the shortest hemostatic time among the four cryogels (*P* < 0.05).

Military is now using Combat Gauze as their gold standard for packing. Thus, the hemostatic capacity of QCSG/CNT0 and QCSG/CNT4 were further evaluated with Combat Gauze as the control group using a standardized circular liver bleeding model (Supplementary Fig. 11). Both cryogel QCSG/CNT0 and cryogel QCSG/CNT4 presented significantly reduced blood loss than Combat Gauze group by employing a standardized circular liver bleeding model (*P* < 0.01), suggesting better hemostatic capacity of the cryogel hemostatic agents than Combat Gauze.

The above results demonstrated that all of the four cryogels showed excellent hemostatic capacity, which were much better than gauze and commercial gelatin hemostatic sponge. Moreover, both QCSG/CNT0 and QCSG/CNT4 presented better hemostatic ability than commercial Combat Gauze. The introduction of appropriate amount of CNT also contributed to enhancing the hemostatic ability of QCSG/CNT cryogels, especially for cryogel QCSG/CNT4. During hemostasis process, both blood cell and platelet activation play important roles^56–58^. Especially, recent study showed that the aggregation of blood cells can change them into a polyhedron shape that can form a perfect seal^3,57^. Platelet adhesion will activate signaling pathways that lead to thromboxane A2 formation and secretion of platelet granule contents, which substances can cause the formation of fibrinogen receptor from integrin and glycoprotein IIb/IIIa leading to platelet aggregation^59^. Then, on the surface of activated platelets, coagulation is accelerated and thrombin is generated, which promotes the hemostatic process^59^. QCSG/CNT cryogels were prepared mainly based on QCSG, which is a chitosan derivative with a large number of positive-charged quaternary ammonium groups. The positive-charged amino groups and quaternary ammonium groups on the cryogel surface would significantly interact with blood cells or platelets via electrostatic interaction to adhere blood cells, platelets, and plasma fibronectin to induce irregular blood cell aggregation, platelet activation, and clot formation^3,31–33^. On the other hand, chitosan’s natural property can promote thrombin generation by shortening the lag time and increasing the maximal values at the later hemostasis^31^. Besides, CNT can interact with platelets, thereby triggering platelets activation and the release of platelet membrane microparticles activated by inducing extracellular Ca^2+^ influx^60,61^. Furthermore, the shape-fixed cryogels with almost closed pores can rapid absorb the plasma and concentrate the blood to entrap aggregated hemocytes, which might immediately form blood-clotting on the material surface^53^. Also, the porous structure and good degree of swelling of the cryogels will improve the adsorption ability for platelets and blood cells to achieve a rapid hemostatic effect^52^. Thus, a synergistic effect of chitosan’s hemostatic nature, strong electrostatic interaction between positive-charged quaternary ammonium groups and blood components, CNT’s platelet activation, high blood absorption capacity, rapid plasma absorbing, and blood-concentrating effects all contributed to the excellent hemostasis performance of CNT-reinforced shape memory cryogels.

### In vivo hemostasis for lethal noncompressible hemorrhage

Uncontrolled hemorrhage leads to more than 30% of trauma deaths, which has been a significant concern of military and civilian trauma centers across the world^7,63,64^. Especially, traditional hemostatic agents present less effective for small and deep wounds with irregular shape, which often cause noncompressible hemorrhage, such as wounds incurred by small-caliber firearms and improvised explosive devices in the battlefield^6^. In this study, we developed injectable CNT-reinforced shape memory nanocomposite cryogel hemostatic dressings with robust mechanical strength, rapid blood-triggered shape recovery, high blood uptake capacity, and rapid blood absorption speed for lethal noncompressible hemorrhage hemostasis, which was evaluated by employing a rabbit liver volume defect model (Fig. 7g–i, Supplementary Note 7). When applying gelatin hemostatic sponge D1 (with a diameter similar to shape-fixed cryogels groups (with diameters of 4 mm) but slightly smaller than wound’s diameter (5 mm)) into the liver defect hole, the bleeding was significantly reduced, which presented a blood loss of 7.1 g (*P* < 0.001). The hemostatic time was also reduced to 8.6 min when compared to blank group (*P* < 0.001). Furthermore, when increasing the gelatin hemostatic sponge’s diameter to 6 mm, compared with gelatin hemostatic sponge D1, blood loss for gelatin hemostatic sponge D2 was continuously reduced to 2.6 g (*P* < 0.05). The hemostatic time was also reduced to 3.9 min when compared to gelatin hemostatic sponge D1 (*P* < 0.05). Interestingly, when injecting QCSG/CNT0 cryogel into the liver defect hole, it presented less blood loss of 0.8 g than both the two gelatin sponges (*P* < 0.05) and blank group (*P* < 0.001). Moreover, cryogel QCSG/CNT4 showed continually reduced blood loss of 0.2 g, which was also significantly less than blank group (*P* < 0.001) and the two gelatin sponges (*P* < 0.05). Furthermore, all the rabbits in the four hemostatic agent groups were alive after a week observation and there was no significant difference in hemostatic time for the gelatin hemostatic sponge D2, QCSG/CNT0 and QCSG/CNT4 (*P* > 0.05). However, gelatin hemostatic sponge D1 showed significantly longer hemostatic time than the other three hemostatic agents (*P* < 0.05). The photographs in Supplementary Fig. 12 were also consistent with the above quantitative data. Compared to gelatin hemostatic sponge without shape memory property (Supplementary Note 8), the shape memory cryogel could be injected into the narrow, deep and irregular wound in a shape-fixed state (Fig. 7j, Supplementary Movie 1 and Supplementary Movie 2), and they would then immediately absorb the blood, concentrate the blood, and instantly recover their initial volume to fill the irregular wound site and keep robust mechanical strength. These properties of the cryogels not only accelerated the blood-clotting speed but also severed as effective physical barriers to stop the bleeding. Especially, CNT-reinforced QCSG/CNT4 had better mechanical property to provide a stronger physical barrier, and the hemostatic ability of CNT further enhanced the blood-clotting ability of the CNT-contained cryogel, thus promoting the liver volume defect hemostasis, indicating that QCSG/CNT4 had great potential for lethal noncompressible hemorrhage application. For clinic application, the cryogels can be fabricated into different shapes to meet the practical applications (Supplementary Note 9). Furthermore, X-ray device can be used to further confirm the complete removal of the CNT-contained cryogel after in vivo application (Supplementary Fig. 13).

### In vivo wound healing performance of the cryogels

**Fig. 8:**
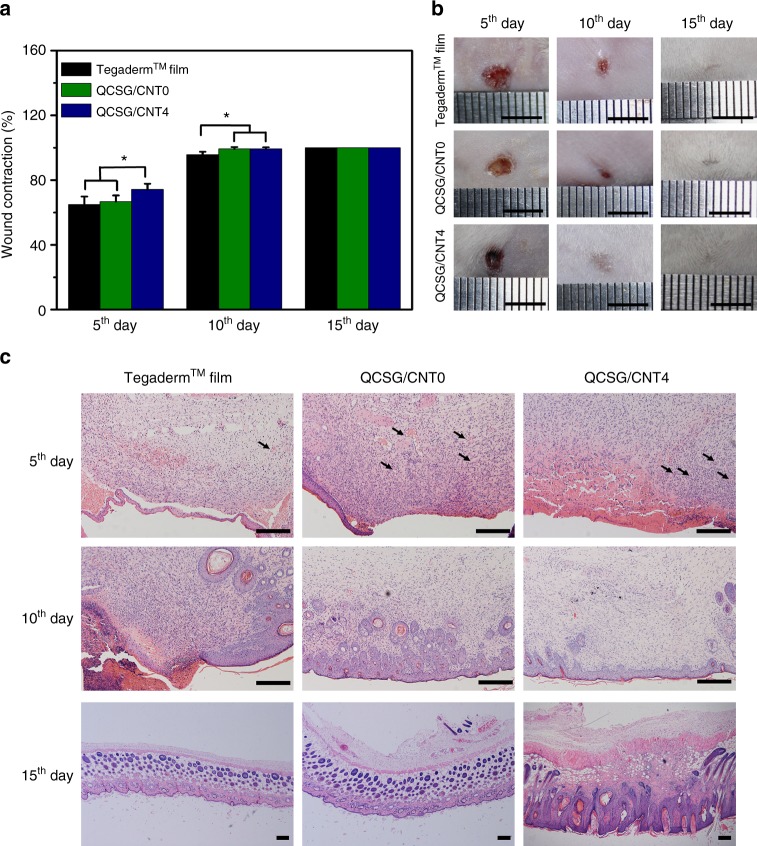
In vivo wound healing performance of the cryogels. **a** Wound contraction for Tegaderm^TM^ film, QCSG/CNT0 and QCSG/CNT4. **b** Photographs of wounds at 5th, 10th, and 15th day for Tegaderm^TM^ film, QCSG/CNT0 and QCSG/CNT4. Scale bar: 5 mm. **c** Histomorphological evaluation of wound regeneration for Tegaderm^TM^ film, QCSG/CNT0 and QCSG/CNT4 at 5th, 10th, and 15th day. Smooth and complete epithelium layer was presented in two cryogel groups at 10th day, differing from the wound sites in Tegaderm™ group whose epithelium layer was still incomplete and rough. The wounds in QCSG/CNT4 group had a better formation of hair follicles. All wounds were completely healed and characterized with perfect epithelization at 15^th^ day. *P < 0.05 using Student's *t*-test (two-sided). Scale bar: 5 mm. Error bar indicates s.e.m. (*n* = 5)

Skin composed of dermis, epidermis, and corneum, is one of the electrical signal sensitive tissues and presents conductivity values from 2.6 mS/cm to 1 × 10^−4^ mS/cm varying different skin components, and studies had demonstrated that the conductive dressings were beneficial to wound healing processes^43,65,66^. Thus, the wound healing performance of the conductive cryogels was investigated by in vivo test using a full-thickness skin defect model (Supplementary Note 10). As shown in Fig. 8a–b, after treated for five days, QCSG/CNT4 group showed smaller wound area than Tegaderm™ dressing and QCSG/CNT0 (P < 0.05). However, there was no significant difference between QCSG/CNT0 and Tegaderm™ dressing (*P* > 0.05). When treated for 10 days, both QCSG/CNT4 and QCSG/CNT0 showed significantly enhanced wound contraction ratio than Tegaderm™ dressing (*P* < 0.05), and there was no significant difference between the two cryogels (*P* > 0.05). Besides, some of mice in the two cryogel groups showed 100% wound contraction while there were few mice showing 100% wound contraction in Tegaderm™ dressing group. At 15th day, all the mice in the three groups presented 100% wound contraction.

The histomorphological evaluation was performed to analyze inflammation and vascularization. Low level inflammation in early stage of wound healing process is a promotive factor for wound healing process but severe inflammatory response impairs the tissue in wound sites with inflammatory cells, oxidation, and fibrotic repair. In general, various degree of inflammation infiltration was observed in three different groups. Excessive amounts of inflammatory cells were recruited in wound sites in Tegaderm™ dressing group than the two cryogel groups (Fig. 8c), especially at 5th day. Inflammation was improved along with healing process, and the inflammatory cells were less in three groups at 10th day. Wound in QCSG/CNT4 groups exhibited the least inflammatory infiltration among three groups at 10th day. All wound sites were fully closed at 15th day, and inflammation was not remarkable in three groups.

It is known that vascularization plays an important role in wound healing process. The blood stream promotes fibroblast recruitment to the wound sites and helps to maintain low level of inflammation for anti-bacteria by bringing the macrophages and other monocytes. In addition, vascularization also brings different growth factors into the wound accelerating the healing process. The vascularization in both QCSG/CNT0 and QCSG/CNT4 groups were higher than the Tegaderm™ film group at 5th day (Fig. 8c). More blood vessels were seen in the two cryogel groups than that in Tegaderm™ film group. The vascularization was lessened in following days in all groups. It is concluded that the cryogel groups had a promotive effect on vascularization in the acute stage of wound healing, which helped the wound to heal.

The integrity of epithelium in wound site varied in each group. The enhanced wound healing performance of conductive QCSG/CNT4 dressing than CNT-free QCSG/CNT0 dressing might be attributed to the transfer of electro-signals from conductive cryogel dressing to the wound site, activation of cellular activity and positive effect of CNT on the level of growth factors involved in wound healing process^43^. Based on the above results and discussions, it revealed that both the two cryogels could accelerate wound healing process by promoting vascularization, and QCSG/CNT4 could further enhance the wound healing by balancing the inflammatory infiltration probably attributed to CNT’s effect, suggesting their huge potential as wound dressings.

### In vivo host response of the cryogels

In vivo host response of the cryogels showed a mild inflammatory responses from H&E staining and toluidine blue staining after implantation for 7 and 30 days (Supplementary Fig. 14 and Supplementary Note 11), indicating that these cryogels could be used as biocompatible temporary cryogel hemostatic agents for in vivo application.

## Discussion

We developed a series of CNT-reinforced antibacterial and conductive nanocomposite cryogels as injectable shape memory hemostatic dressings, and demonstrated that they not only promoted the wound healing process in a full-thickness skin defect model but also presented excellent hemostatic effects in mouse liver injury model, mouse-tail amputation model, rabbit liver defect lethal noncompressible hemorrhage model, and a standardized circular liver bleeding model. The cryogels exhibited better hemostatic effect than gauze and gelatin hemostatic sponge in mouse liver injury model and mouse-tail amputation model, and better hemostatic effect than Combat Gauze in a standardized circular liver bleeding model for their good blood-clotting ability, higher blood cell and platelet adhesion and activation. Furthermore, the cryogel QCSG/CNT4 showed better hemostatic capability than QCSG/CNT0 for its optimized properties, and better in vivo wound healing performance than Tegaderm™ film and QCSG/CNT0 for the introduction of CNT. More importantly, QCSG/CNT4 also presented better hemostatic performance than gelatin hemostatic sponge and QCSG/CNT0 in rabbit liver defect lethal noncompressible hemorrhage model. All these results demonstrated that the CNT-reinforced nanocomposite cryogel hemostatic dressings with multiple functions are excellent candidates as hemostatic dressings for lethal noncompressible hemorrhage and wound dressing applications.

## Methods

### Synthesis of GMA functionalized quaternized chitosan

The QCSG was synthesized via one-pot reaction between epoxy groups from glycidyltrimethylammonium chloride (GTMAC) and GMA and amino groups from chitosan. 1 g of chitosan (J&K Chemical, *M*_n_ = 100,000–300,000 Da) was suspended in 36 mL of deionized water, and then 180 µL of glacial acetic acid (Sigma-Aldrich) was added to the suspension. After stirring at 55 °C for 30 min, different molar ratios of GTMAC (Sigma-Aldrich) were added dropwise to the chitosan-glacial acetic acid mixture under continuous stirring, respectively. The molar ratios of GTMAC to amino groups on chitosan backbone were varying from 1:1 to 2:1 and 3:1, respectively (Supplementary Table 1). The reaction mixtures were stirred at 55 °C for 15 h. Then, GMA (Sigma-Aldrich) was added dropwise to the above reaction mixtures with continuous stirring at 55 °C, respectively. The ratio of GMA to amino groups on the pure chitosan backbone was fixed at 0.5:1.0, and the reaction was performed for another 15 h at 55 °C in the dark condition (Supplementary Table 1). After the reaction, the undissolved polymer was removed by centrifuging the mixture at 5692× *g* for 20 min at room temperature. The supernatant liquid was precipitated into pre-cooled acetone to obtain the crude product. For purifying the product, the crude product was dissolved in DI water, and then dialyzed exhaustively (MWCO 3500) against deionized water for three days in the dark condition. The pure product was obtained by lyophilization. The quaternary amination degrees of the three QCSG copolymers were determined by titrating the content of chlorine ion^27^. The chemical structure of QCSG was confirmed by ^1^H NMR (Supplementary Fig. 15) and FT-IR (Supplementary Fig. 2) spectra. The parameters of the synthesized QCSG copolymers were listed in Supplementary Table 1. The chemical structure of QCSG was confirmed by ^1^H NMR (Supplementary Fig. 15) and FT-IR (Supplementary Fig. 2) spectra. The parameters of the synthesized QCSG copolymers were listed in Supplementary Table 1.

### Synthesis of diacrylate functionalized PF127 (PF127-DA)

The PF127-DA was prepared by acrylation of PF127 (poly(ethylene glycol)-*b*-poly(propylene glycol)-*b*-poly(ethylene glycol)) using acryloyl chloride according to reference^13^. A volume of 2.54 g (0.2 mmol) of PF127 (Sigma-Aldrich) and 0.061 g of triethylamine (J&K Chemical) (0.6 mmol) were dissolved in 20 mL anhydrous dichloromethane in an ice bath and then degassed by pouring nitrogen for 20 min. After that, 0.05 mL of acryloyl chloride (0.6 mmol) was slowly injected into the above solution under nitrogen environment. The reaction was performed at room temperature for 24 h. Following the reaction, the solvent was removed by rotational evaporation, and the crude product was dissolved in DI water, and dialyzed exhaustively (MWCO 3500) against deionized water for three days. The pure product was obtained by lyophilization. The chemical structure of PF127-DA was confirmed by ^1^H NMR (Supplementary Fig. 16) and FT-IR (Supplementary Fig. 17) spectra.

### Preparation of QCSG/CNT cryogels

The cryogels were prepared by cryopolymerization of QCSG aqueous solution and PF127-DA/CNT aqueous dispersion at −20 °C using APS/TEMED as redox initiator system. Firstly, the multi-walled CNTs (Nanjing XFNANO Materials Tech Co., the diameter, length, conductivity and special surface area of the CNT are 10–20 nm, 10–30 μm, ≥100 S/cm, and ≥200 m^2^/g, respectively) dispersion was prepared^13^. Briefly, equal weights of CNT and PF127-DA were added into 8 mL of deionized water. Then, the mixture was sonicated in ice bath for 4 h, and another 1 h before use. QCSG was dissolved in DI water to form a 5 wt% of QCSG solution. A volume of 10 mL of QCSG solution was added into the above CNT dispersion solutions or 8 mL of deionized water, respectively, and then sufficiently mixed. Following that, 1 mL of APS (Sigma- Aldrich) solution (100 mg/mL) and 1 mL of TEMED (Sigma-Aldrich) solution (20 µL/mL) were added into QCSG and QCSG/CNT mixtures (pre-cooled in ice bath), and mixed sequentially in an ice bath. After that, the cryogel precursor was transferred into cylindrical mold (with a diameter of 10 mm) and placed in a freezer at −20 °C. The polymerization was allowed to proceed for 18 h, and the resulting cryogels were thawed. The obtained QCSG/CNT cryogels were purified by immersed in DI water for seven days to remove the unreacted polymer and free CNT, and the purified QCSG/CNT cryogels showed no CNT leakage (<0.1% CNT) when further immersed in DI water. Four cryogels with a constant 2.5 wt% QCSG concentration and the CNT concentrations varying from 0 to 2, 4, and 6 mg/mL were prepared in this study as shown in Supplementary Table 2.

### ^1^H NMR spectrum test

The spectra of QCSG and PF127-DA were performed using a Bruker Ascend 400 MHz NMR instrument with deuteroxide and chloroform-d serving as the solvents and internal standards.

### FTIR spectrum test

The spectra of QCSG, PF127, PF127-DA, dried cryogel QCSG/CNT0, and dried cryogel QCSG/CNT2 were recorded in the range of 4000–650 cm^−1^ by employing a Nicolet 6700 FT-IR spectrometer (Thermo Scientific Instrument).

### UV-vis spectrum test

The spectra of the supernatants of CNT aqueous dispersion, PF127-DA/CNT aqueous dispersion and PF127-DA/CNT/QCSG aqueous dispersion after placed at room temperature for 24 h were recorded using a spectrophotometer (Lambda 35, PerkinElmer). The CNT aqueous dispersion and PF127-DA/CNT aqueous dispersion were prepared by sonicating CNT/DI water mixture and PF127/CNT/DI water mixture in ice bath for 4 h, respectively. PF127-DA/CNT/QCSG aqueous dispersion was prepared by fine mixing PF127-DA/CNT aqueous dispersion and QCSG solution. The three aqueous dispersions had the same CNT concentration of 2 mg/mL, while QCSG was 12.5 mg/mL.

### Scanning electron microscope (SEM) morphology observation

The morphologies of the freeze-dried cryogels (including shape-free state, shape-fixed state, and recovered shape from shape-fixed state) were observed using a field emission scanning electron microscope (FEI Quanta FEG 250). Before observation, the surface of the cryogels was sprayed with a gold layer.

### Swelling ratio test

The cryogels equilibrated in DI water at room temperature were firstly weighted (*W*_E_). Then, they were lyophilized and weighted again (*W*_O_). The swelling ratio (SR) was calculated as:

1 $${\mathrm{SR = }}\left( {{{W}}_{\mathrm{E}}{{ - W}}_{\mathrm{O}}} \right){{/W}}_{\mathrm{O}}{\mathrm{ \times 100\% }}$$

### Cryogel conductivity and impedance curve

The resistance of the cryogels under both swollen state and lyophilized state was measured by an Agilent B2900A digital 4-probe tester with a current of 1 mA and a linear probe head (1.0 mm space). All the cryogels were washed with DI water to remove the initiator and unreacted polymer. The conductivity of the cryogels can be calculated using the equation:

2 $${\it{\sigma }}{\mathrm{ = 1/\rho }}$$

where *ρ* was resistance and *σ* was conductivity. Each sample was measured under small compressive strain, and the average results of three values measured under positive current and three data obtained under negative current were taken. Furthermore, the impedance curves of dried cryogel QCSG/CNT4 and dried cryogel QCSG/CNT0 were performed by using electrochemical workstation (CHI660E electrochemical analyzer) with the frequency from 10^2^ to 10^6^ Hz.

### X-ray detectability of cryogel

The X-ray detectable nanocomposite cryogel hemostatic was prepared by gluing a X-ray detectable line to the cryogel surface. Then, the Micro-CT analysis was performed on a 3D microfocus X-ray microcomputed tomography system (Y.CHEETAH*, YXLON) to confirm the X-ray detectability of the X-ray detectable line contained nanocomposite cryogel hemostatic. The nanocomposite cryogel without X-ray detectable line was used as a control.

### Injectability of the cryogel

The cryogels were prepared with a free shape diameter of 5 mm. Then, the shape of the cryogels was fixed by removing the free water within the cryogels’ matrix. After that, the shape-fixed cryogels were loaded into an injector (with a blunt nozzle inner diameter of 1.5 mm), and the cryogels were injected into the water to recover their shapes. The injection processes were videotaped and photographed.

### Rheological properties of the cryogel

The rheological test was performed by employing a TA rheometer (DHR-2) using two different methods. The cryogels were cut into disk shapes with diameters of 12 mm. The oscillation-frequency experiments were conducted under a constant strain of 1% and varying the shear rate from 0.1 rad/s to 100 rad/s at 25 °C. The strain amplitude sweep tests (*γ* = 0.01%-100%) of the cryogels (with diameters of 12 mm) were also performed using constant frequency of 10 rad/s at 25 °C.

### Minimum inhibitory concentration assay of copolymers

The copolymers’ MICs for both *E. coli* (ATCC 8739) and *S. aureus* (ATCC 29213) were evaluated according to our previous report^27^. Briefly, the copolymers were dissolved in deionized water and then diluted with MH broth using a twofold dilution method. 100 μL of bacterial suspension (10^4^–10^5^ CFU mL^−1^) was added to the 96-well plate with a series 100 μL of twofold dilution copolymers MH broth solutions and then homogeneously mixed by pipetting. After that, the 96-well clear plate (Costar) was placed in an incubator at 37 °C for 18 h. MH broth without inoculum was used as the negative control, while MH broth with inoculum served as positive control. At the end of the time, the absorbance of the solutions in the 96-well plate was read using a microplate reader (Molecular Devices) at 600 nm. The copolymers’ MICs were defined as the minimum concentrations, which inhibited over 90% of bacteria growth.

### Mechanical properties of the cryogels

The mechanical properties of the cryogels were evaluated by compression test and cyclic compression test employing a TA rheometer (DHR-2) at room temperature. The cryogel sample was prepared as cylindrical shape with a height of 10 mm and diameter of 8 mm. The maximal compression strain of 93% was chosen to perform the compression-strain test with a strain speed of 100 µm/s. For the cyclic compression test, a drop of water was added around the cryogel sample on the platform before the test, and a 40%, 60%, and 80% compression strain were also applied to perform the cyclic compression test. The compression strain was firstly performed up to the pre-set strain and then released to 0% strain with constant compression and release strain rate of 100 µm/s, which were cycled for 10 times. Besides, a cyclic compression test was performed on cryogel QCSG/CNT0 and cryogel QCSG/CNT4 for 100 cycles with the compression strain up to 80% at a strain speed of 100 µm/s, and then the tested QCSG/CNT4 was further immersed in DI water at 37 °C with a 100 rpm shaking speed for another 24 h. The diffused CNT was determined by UV-vis^13^.

When injecting or implanting the cryogel in bleeding site or wound, the cryogel would bear continuous and dynamic force loading. In order to evaluate the CNT stability of the cryogel when bearing continuous and dynamic compression, we performed two tests to simulate the in vivo application situations. First, we soaked cryogel QCSG/CNT4 (with a height of 10 mm and diameter of 8 mm) in 3 mL DI water and then performed a dynamic compression test with the compression strain up to 50% at a strain speed of 50 µm/s for 6 h. After the test, the released CNT in the DI water was determined by UV-vis^13^. Furthermore, the tested cryogel QCSG/CNT4 was also immersed in 3 mL DI water at 37 °C with a 100 rpm shaking speed for another 24 h, and then the released CNT was also determined by the UV-vis.

### Shape memory behavior of the cryogels

The shape memory property of the cryogels was evaluated according to reference^13^. The as-prepared cryogel (with initial length of 10 mm (*L*_1_)) was compressed to 80% strain at a strain rate of 100 µm/s and then hold at this strain for 1 min. Then the water squeezed out from the cryogel was absorbed away using paper completely and the compressed gauge length was set as *L*_2_*.* After that, the sample was free of any load for 5 min, a fixed gauge length was measured as *L*_3_. Then the sample was soaked in water for re-hydration for 1 min, and the recovery gauge length was measured as *L*_4_. The test was performed employing a TA rheometer. The *L*_2_ was measured by instrument automatically, while *L*_1_, *L*_3_, and *L*_4_ were measured manually by fine using rheometer’s software to adjust gap distance. The test was cycled for five times. The shape memory fixity ratio and recovery ratio were calculated according to following equations:

3 $${\mathrm{Maximum}}\;{\mathrm{compressive}}\;{\mathrm{strain:}}\;\varepsilon _{\mathrm{m}}\,{\mathrm{ = }}\,\left( {{{L}}_{\mathrm{1}}{{- L}}_{\mathrm{2}}} \right){{/L}}_{\mathrm{1}} \times {\mathrm{100\% }}$$

4 $${\mathrm{Fixed}}\;{\mathrm{strain:}}\;\varepsilon _{\mathrm{u}}\,{\mathrm{ = }}\,\left( {{{L}}_{\mathrm{1}}{{ - L}}_{\mathrm{3}}} \right){{/L}}_{\mathrm{1}} \times {\mathrm{100\% }}$$

5 $${\mathrm{Recovery}}\;{\mathrm{strain:}}\;\varepsilon _{\mathrm{p}}\,{\mathrm{ = }}\,\left( {{{L}}_{\mathrm{4}}{{- L}}_{\mathrm{3}}} \right){{/L}}_{\mathrm{1}} \times {\mathrm{100\% }}$$

6 $${\mathrm{Strain}}\;{\mathrm{fixity}}\;{\mathrm{ratio:}}\;{{R}}_{\mathrm{f}}\,{\mathrm{ = }}\,\varepsilon _{\mathrm{u}}{\mathrm{/ }}\varepsilon _{\mathrm{m}} \times {\mathrm{100\% }}$$

7 $${\mathrm{Strain}}\;{\mathrm{recovery}}\;{\mathrm{ratio}}\;\,{\mathrm{ = }}\,\varepsilon _{\mathrm{p}}{\mathrm{/ }}\varepsilon _{\mathrm{u}} \times 100\% \ = \ (L_4-L_3) {\mathrm {/}} (L_1-L_3) \times100\% .$$

### Volumetric expansion ratios of the cryogels

Before fixing the cryogel shape, the shape-free diameter (*D*_1_) and length (*L*_1_) of columniform cryogel were tested. After that, the free water in the columniform cryogel was squeezed out to obtain the shape-fixed cryogel. And then the diameter (*D*_2_) and length (*L*_2_) of the shape-fixed cryogel was further determined. The volumetric expansion ratio was calculated using the following equation:

8 $${\mathrm{Volumetric}}\;{\mathrm{expansion}}\;{\mathrm{ratio = }}\frac{{\left( {\frac{{D1}}{2}} \right)^2 \times L1}}{{\left( {\frac{{D2}}{2}} \right)^2 \times L2}}$$

### Hemolytic activity assay of the cryogels

For hemolytic activity assay, the erythrocytes were obtained by centrifuging (at 116× *g*) the mouse blood for 10 min. DPBS was used to wash the obtained erythrocytes for three times, and then the purified erythrocytes was further diluted to a final concentration of 5% (v/v). After that, the dried cryogel was smashed into homogenate by employing a tissue grinder, and four cryogel dispersion liquids (with the concentrations varying from 5 to 2.5, 1.25, and 0.625 mg/mL) were prepared. 0.5 mL of the cryogel dispersion liquid and 500 µL of erythrocyte suspension (5% (v/v)) were added into a 2-mL tube, and then they were gently mixed by pipetting. After placed at 37 °C for 1 h, all the samples were centrifuged at 116× *g* for 10 min. A volume of 500 µL of the supernatants was carefully transferred into new tubes, respectively, and then the supernatants were further centrifuged at 11617× *g* for 10 min allowing exhaustively to remove the cryogel particles. The obtained supernatants were transferred into a new 96-well clear plate. The absorbance of the solutions at 540 nm was read using a microplate reader (Molecular Devices). 0.1% Triton X-100 served as the positive control and DPBS served as the negative control. The hemolysis percentage of the cryogels was calculated using the equation:

9 $${\mathrm{Hemolysis}}\;\left( {\mathrm{\% }} \right){\mathrm{ = }}\left[ {\left( {{\mathrm{Ap-Ab}}} \right){\mathrm{/}}\left( {{\mathrm{At-Ab}}} \right)} \right]{\mathrm{ \times 100\% }}$$

where Ap was the absorbance value of supernatant from the cryogel groups, At was the absorbance value of the Triton X-100 positive control and Ab was the absorbance value of DPBS. Each group contains three repeats.

### Cytotoxicity test of the cryogels

The cytotoxicity of the cryogels on L929 (ATCC CCL-1, the L929 cell was purchased from the Shanghai Cell Bank of the Chinese Academy of Sciences) cell was evaluated using two methods including a leaching pattern and a direct contact test between cryogel and cells. The cryogel was cut into disks with 8 mm diameter and 5 mm thickness and sterilized by immersing in 75% alcohol. The dulbecco’s modified eagle medium (DMEM) (Gibco) supplemented with 10% fetal bovine serum (Gibco), 1.0 × 10^5^ U/L penicillin (Hyclone) and 100 mg/L streptomycin (Hyclone) was used as the complete growth medium. L929 cells were seeded in 48-well plate at a density of 25,000 cells/well. After cultured for 24 h, the cryogel disks equilibrated in the complete growth medium were introduced into the wells and the culture medium level was slightly lower than the cryogel upper surface by removing the excess medium to allow the fine contact between cell and cryogel. The cell viability under the cryogel was evaluated by alamarBlue^®^ assay and LIVE/DEAD^®^ Viability/Cytotoxicity Kit assay after cultured for 24 h. The cryogel disks and medium were removed and 20 μL of alamarBlue^®^ reagent in 200 μL complete growth medium was then added into each well. The plate was incubated for 4 h in a humidified incubator containing 5% CO_2_ at 37 °C. After that, 100 μL of the medium in each well was transferred into a 96-well black plate (Costar). Fluorescence was read using 560 nm as the excitation wavelength and 600 nm as the emission wavelength using a microplate reader (Molecular Devices) according to the manufacturer’s instructions. Cells seeded on TCP without cryogel disc served as the positive control group. The tests were repeated four times for each group. Cell adhesion and viability were observed under an inverted fluorescence microscope (IX53, Olympus). The cell viability was evaluated by alamarBlue^®^ assay after cultured for 24 h.

For the leaching pattern assay, the sterilized cryogel extract solutions with the cryogel weights varying from 20 to 15, 10, and 5 mg/mL in the culture medium were prepared by immersing the dried cryogels in medium for 24 h at 37 °C with a shaking speed of 100 rpm. L929 cells were seeded in 96-well plate at a density of 10000 cells/well, and pre-cultured for 24 h before replacing the culture medium with the fresh medium containing a series of different concentrations of cryogel extract solutions. After cultured for 24 h, the cell viability was evaluated by alamarBlue^®^ assay. The medium was removed and 10 μL of alamarBlue^®^ reagent in 100 μL complete growth medium was then added into each well. The plate was incubated for 4 h in a humidified incubator containing 5% CO_2_ at 37 °C. After that, 100 μL of the medium in each well was transferred into a 96-well black plate (Costar). Fluorescence was read using 560 nm as the excitation wavelength and 600 nm as the emission wavelength using a microplate reader (Molecular Devices) according to the manufacturer’s instructions. Cells seeded on TCP without extract solution served as the positive control group. The tests were repeated four times for each group.

### Whole-blood clotting of the cryogels

The whole-blood clotting of the cryogels were tested according to the literature^4,54^. The cryogel was cut into cylindrical cryogel with a height of 5 mm and a diameter of 8 mm, and then the cylindrical cryogels were formed into shape-fixed situation. A volume of 50 µL of recalcified whole-blood solution (0.2 M CaCl_2_, 10 mM in the blood) was added onto the pre-warmed cryogels (37 °C) in polypropylene tubes, respectively. Then, the tube was incubated at 37 °C for 30 s, 60 s, 90 s, 120 s, and 150 s, respectively. The gauze and gelatin hemostatic sponge were used as control groups. After the pre-set period, 10 mL of DI water was gently added to release unbound blood without disturbing the clot. The absorbance of the supernatant was recorded at 540 nm by using a microplate reader (Molecular Devices). Three to five replicates were performed. The absorbance of 50 μL of recalcified whole-blood in 10 mL DI water was used as the reference value (negative control). The blood-clotting index (BCI) was calculated using equation:

10 $${\mathrm{BCI}}\;\left( {\mathrm{\% }} \right){\mathrm{ = }}\left[ {\left( {{{I}}_{\mathrm{s}}{{ - I}}_{\mathrm{o}}} \right){\mathrm{/}}\left( {{{I}}_{\mathrm{r}}{{ - I}}_{\mathrm{o}}} \right)} \right]{\mathrm{ \times 100\% }}$$

where *I*_s_ represented the absorbance of sample and *I*_r_ represented the absorbance of the reference value.

### Blood cell and platelet adhesion on the cryogels

The blood cell and platelet adhesion assay was performed according to reference^52^. The cryogel sample was cut into disks with a height of 5 mm and a diameter of 8 mm, which were further immersed into DPBS for 1 h at 37 °C. After that, the ACD-whole-blood was dropwise introduced onto the cryogel disks and then placed at 37 °C for 5 min. Platelet-rich plasma (PRP) was obtained by centrifuging the ACD-whole-blood at 116× *g* for 10 min. Then, PRP was dropwise introduced onto the cryogel disks and further placed at 37 °C for 1 h. At the end of the time, all the test samples were washed by DPBS for three times to remove the physically adhered blood cell and platelet. Then the samples were fixed using a 2.5% glutaraldehyde for another 2 h. After that, blood cells and platelets in the samples were gradually dehydrated using 50%, 60%, 70%, 80%, 90%, and 100% ethanol solution with time interval of 10 min. The dried samples were observed using SEM.

### Photothermal effect of the cryogels

In order to elucidate the cryogels’ photothermal effect, the as-prepared cryogel was cut into disks (a diameter of 8 mm and a height of 5 mm) and then exposed to an NIR laser (MDL-III-808nm-1000mW, Changchun New Industries Optoelectronics Tech Co., Ltd.) at a power density of 1.4 W/cm^2^ for 20 min. The heat maps and temperature profiles of the cryogels were recorded using an infrared (IR) thermal camera. Besides, the heat maps and temperature profiles of the QCSG/CNT4 were recorded at power density varying from 0.6 to 0.9, 1.1, and 1.4 W/cm^2^, respectively.

### NIR-stimulus responsive release behavior of ibuprofen

In order to encapsulate ibuprofen into the QCSG/CNT0 and QCSG/CNT4, 2 mg of ibuprofen was added into 0.4 mL DPBS or 0.4 mL DPBS containing 4 mg of CNT and 4 mg of PF127-DA, respectively. Then, the mixture was sonicated in ice bath for 4 h. 0.5 mL of 5 wt% of QCSG solution was added into the above 0.4 mL of PF127-DA/CNT/ibuprofen DPBS dispersion or 0.4 mL of ibuprofen DPBS dispersion, respectively, and then sufficiently mixed. After that, 50 µL of ammonium persulfate (APS) (100 mg/mL) and 50 µL of tetramethylethylenediamine (TEMED) (20 µL/mL) were added into the QCSG or QCSG/CNT mixtures (pre-cooled in ice bath) and mixed sequentially in an ice bath. Then the cryogel precursor was transferred into cylindrical mold (with a diameter of 10 mm) and placed in a freezer set to −20 °C. After reaction for 18 h, the resulting ibuprofen loaded QCSG/CNT0 and QCSG/CNT4 cryogels were thawed for drug controlled release test. The drug loaded cryogels (500 µL) in tubes with 1.4 mL of DPBS (pH=7.4, 0.01 M) were placed at 37 ^o^C with a shaking speed of 100 rpm to perform the spontaneous drug release profiles. For the NIR triggered ibuprofen release from QCSG/CNT4, the cryogels (500 µL) in tubes with 1.4 mL of DPBS (pH=7.4, 0.01 M) were irradiated with 808 nm NIR light (1.4 W/cm^2^) over a period of 10 min. The ibuprofen free cryogels were used as blank controls. At predetermined time intervals, 1 mL of the release buffer was taken out for further analysis. Subsequently, 1 mL of fresh buffer was added to the tubes in order to maintain the constant volume. The concentrations of the drugs were analyzed by the UV–vis spectrophotometer (PerkinElmer Lambda 35). The *λ*_max_ of ibuprofen was 264 nm.

### NIR irradiation enhanced antibacterial test of cryogels

The cryogel QCSG/CNT4 disks (with a diameter of 8 mm and thickness of about 5 mm) were sterilized by immersing the samples in 75% alcohol, and then equilibrated with sterilized Dulbecco’s phosphate-buffered saline (DPBS). A volume of 10 µL of bacterial suspension in sterilized DPBS (10^8^ CFU mL^−1^) was added onto the surface of the swollen cryogel disks (QCSG/CNT0 and QCSG/CNT4). Then, the cryogel was exposed to NIR laser light (808 nm, 1.4 W/cm^2^) for varying periods from 0 to 1, 3, 5, 10, and 20 min, respectively. A volume of 10 µL of bacterial suspension (10^8^ CFU mL^−1^) suspended in 200 µL of DPBS was used as a negative control, which was also exposed to NIR laser light (808 nm, 1.4 W/cm^2^). After allowing all the groups contact with bacteria for 20 min, 1 mL of sterilized DPBS was added into each well to re-suspend any bacterial survivor. Then, 10 µL of the above bacterial survivor resuspension was added onto agar plate, the colony-forming units on the agar plate were counted after incubated for 18 to 24 h at 37 °C. The tests were repeated three times for each group and the results were expressed as log 10 CFU using equation:

11 $${\mathrm{Log}}\;{\mathrm{Reduction}} = {\mathrm{Log}}\;{\mathrm{cell}}\;{\mathrm{count}}\;{\mathrm{of}}\;{\mathrm{control}} - {\mathrm{Log}}\;{\mathrm{survivor}}\;{\mathrm{count}}\;{\mathrm{on}}\;{\mathrm{cryogel}}.$$

### In vivo hemostatic performance of the cryogels

The hemostatic ability of the cryogels were evaluated by both mouse liver trauma model^43^ and mouse-tail amputation model^54^. All animal studies were approved by the animal research committee of Xi’an Jiaotong University. For mouse liver trauma model, the mice (Kunming mice, 5–6 week-old, weight of 32−38 g, female) were randomly and equally divided into seven groups. The animals were anesthetized by injecting 10 wt% chloral hydrate and fixed on a surgical corkboard. The liver of the mouse was exposed by abdominal incision, and serous fluid around the liver was carefully removed to prevent inaccuracies in the estimation of the blood weight obtained by the hemostatic samples. A pre-weighted filter paper on a paraffin film was placed beneath the liver. Bleeding from the liver was induced using a 16 G needle with the corkboard tilted at about 30°. The pre-weighted gauze, gelatin hemostatic sponge, and shape-fixed cryogels were immediately applied onto the bleeding site, respectively. No treatment after pricking the liver was used as control group. The data of bleeding time and blood loss were recorded during the hemostatic process. Each group contains ten mice.

For the mouse-tail amputation model, the mice (Kunming mice, 5–6-week-old, weighing 32−38 g, female) were randomly and equally divided into seven groups. The animals were anesthetized by injecting 10 wt% chloral hydrate (0.3 mL per 100 g weight of animal) and fixed on a surgical corkboard. Fifty percent length of the tail was cut by surgical scissors. After cutting, the tail of the mouse was placed in air for 15 s to ensure normal blood loss. Then the wound was covered with the pre-weighted gauze, hemostatic sponge, and shape-fixed cryogels under slight pressure. The data of bleeding time and blood loss were recorded during the hemostatic process. The wound without treatment was used as control group. Each group contains ten mice.

### In vivo lethal noncompressible hemorrhage hemostasis test

New Zealand White rabbit liver volume defect was used as a lethal noncompressible hemorrhage hemostasis model to evaluate the hemostatic capacity of the cryogels. The shape-fixed cryogel was injected into the liver defect hole to rapidly stop the bleeding and then cause the hemostasis. The shape of the sterilized cryogels (with diameter of 8 mm and height of 8 mm) was fixed as section 2.6 described, and the diameter of the shape-fixed cryogels was 4 mm. Then the shape-fixed cryogel was loaded into syringe (with inner diameter of 4 mm and external diameter of 5 mm) for further in vivo injection. Two types of gelatin hemostatic sponges (with a height of 8 mm and diameters of 4 mm and 6 mm, respectively) were used as control groups. New Zealand White rabbits (male, ∼2.5 kg) were randomly and equally divided into five groups. The animals were fixed on the surgical corkboard and then 10% chloral hydrate was injected into rabbits enterocoelia to anesthetize them (0.5 ml per 100 g). Following that, the rabbit underwent an abdominal incision to expose the liver, the serous fluid around the liver was carefully removed, and then a columniform liver volume defect (with a diameter of 5 mm and height of 5 mm) was made in right lobe using biopsy needle (inner diameter of 5 mm) and surgical scissors. Free bleeding was allowed for 30 s and then the cryogel was immediately injected into the defect hole or gelatin hemostatic sponge was immediately inserted into the defect hole. During the hemostatic process, the weighed gauze was used to absorb the flowing blood. The hemostatic time, blood loss and life state were recorded accordingly. Each group was repeated for five times. The animal experiments were approved by the animal research committee of Xi’an Jiaotong University.

### Hemostatic test on a standardized liver bleeding model

The shape of the sterilized cryogels (with diameter of 12 mm and height of 8 mm) was fixed by compressing and removing the free water. The diameter of the shape-fixed cryogels was about 16 mm. Thirty-two layers of Combat Gauze with a diameter of about 8 mm were used as a control group. New Zealand White rabbits (male, ∼2.5 kg) were randomly and equally divided into four groups. The animals were fixed on the surgical corkboard and then 10% chloral hydrate was injected into rabbits enterocoelia to anaesthetize them (0.5 ml per 100 g). Following that, the rabbit underwent an abdominal incision to expose the liver, the serous fluid around the liver was carefully removed. A plastic disc (with a diameter of 12 mm) with biological glue was glued to the right lobe of the rabbit and then a circular surface defect (with a diameter of 12 mm) was made in the right lobe by cutting the liver along the inside of the disc. The hemostatic agents were immediately applied onto the defect with slight pressure for 15 min after creating the liver surface defect. After the performance, the blood loss was recorded accordingly. The group without any treatment was used as blank group. Each group contains six rabbits. The animal experiments were approved by the animal research committee of Xi’an Jiaotong University.

### In vivo wound healing test

The animal experiments were approved by the animal research committee of Xi’an Jiaotong University. Mice weighing 32–38 g and 5–6-week-old were used for studies and randomly and equally divided into three groups. The mice were acclimatized for one week before surgery groups. For the surgery part, all procedures were performed under aseptic condition. After standard anesthesia procedure with intraperitoneal injection of chloral hydrate (0.3 mg/kg body weight), the dorsal region of mouse above the tail but below the back was shaved to prepare for surgery. Two full-thickness wounds with 6 mm diameter were created on either side of the midline. One group was dressed by Transparent Film Dressing Frame Style (3 M Health Care, USA) and QCSG/CNT0 + Transparent Film Dressing, one group was dressed by Transparent Film Dressing and QCSG/CNT4 + Transparent Film Dressing, and the other group was dressed by QCSG/CNT0 cryogel + Transparent Film Dressing and QCSG/CNT4 cryogel + Transparent Film Dressing. Each group contained five mice. For wound area monitoring, on the 5th, 10th, and 15th day, the mice were performed standard anesthesia procedure with intraperitoneal injection of chloral hydrate, then the wound area was measured by tracing the wound boundaries on plotting papers.

Wound regeneration (%) was calculated using the equation:

12 $${\mathrm{Wound}}\;{\mathrm{contraction = (area}}\;{\mathrm{(0}}\;{\mathrm{day) - area}}\;\left( {{{n}}\;{\mathrm{day}}} \right){\mathrm{)/(area}}\;{\mathrm{(0}}\;{\mathrm{day)) \times 100\% }}$$

### Histological analysis

For evaluation of epidermal regeneration and inflammation in wound area, samples collected on 5th, 10th, and 15th day were fixed with 4% paraformaldehyde for 1 h, then embedded in paraffin, cross sectioned to 40-μm thick slices, and then stained with Haematoxylin-Eosin (Beyotime, China). All slices were analyzed and photo-captured by microscope (IX53, Olympus, Japan).

### In vivo host response evaluation of the cryogels

The in vivo host response evaluation of the cryogels was performed as we previously reported^67^. All of the procedures of animal experiments were performed according to the guidelines established by the animal research committee of Xi’an Jiaotong University. Before implantation, all of the materials were cut into the same shape and size (diameter of 8 mm and height of 2 mm), sterilized with 75% ethanol, and rinsed in phosphate-buffered saline (PBS) overnight. Female Sprague Dawley rats (200−250 g in weight, 7–8 weeks) were generally anesthetized, and a small incision was made on the same area on the back of each rat. The testing articles were placed subcutaneously into the incision, and the skin was closed following the placement. The rats returned to their own cage and permitted free access to food and water after they recovered from anesthesia. After surgery for 7 days and 30 days, the animals were sacrificed and implanted materials were excised with the adjacent tissues. After excision, the material-tissue compounds were embedded in paraffin, sectioned (3 μm), and mounted onto slides. The acute inflammatory response and chronic inflammatory response were evaluated from both hematoxylin and eosin (H&E) staining and toluidine blue (TB) staining. After staining, the slides were observed by microscope and the images were analyzed using Image Pro Plus software.

### Statistical analysis

The experimental data were analyzed using Student’s *t*-test. *P* value < 0.05 was considered statistical significance. Results are expressed as mean ± standard error (s.e.m) for animal studies while the other results are expressed as mean ± standard deviation (s.d.)^62^. The sample size for the animal studies was validated by Gpower3 software using the post-hoc power analysis for a two-tailed *t*-test^68,69^. The effect size index was calculated by the equation:

13 $$d = \frac{{|\mu 1 - \mu 2|}}{{\sqrt {0.5 \times (\sigma 1^2 + \sigma 2^2)} }}$$

that made the power (1 − *β* error probability) ≥ 0.8 for sample size of 10 in mice hemostatic experiments, for sample size of five in rabbit hemostatic experiments and for sample size of five in mice wound healing experiments. For mice hemostatic experiments, average blood losses were *µ*1 and *µ*2 from QCSG/CNT2 treated mice liver group and QCSG/CNT6 treated mice liver group, and *σ*1 and *σ*2 were standard deviations of those from QCSG/CNT2 and QCSG/CNT6 treated mice liver groups, respectively. For rabbit hemostatic experiments, average blood losses were *µ*1 and *µ*2 from gelatin sponge D2 treated rabbit liver group and QCSG/CNT0 treated rabbit liver group, and *σ*1 and *σ*2 were standard deviations of those from gelatin sponge D2 and QCSG/CNT0 treated rabbit liver groups, respectively. For mice wound healing experiments, average wound contraction percentages were *µ*1 and *µ*2 from QCSG/CNT0 treated mice skin wound group and QCSG/CNT4 treated mice skin wound group, and *σ*1 and *σ*2 were standard deviations of those from QCSG/CNT0 and QCSG/CNT4 treated mice skin wound groups, respectively.

### Data availability

The data that support the findings of this study are available from the corresponding author upon reasonable request.

## Electronic supplementary material

Supplementary Information

Description of Additional Supplementary Files

Supplementary Movie 1

Supplementary Movie 2

Supplementary Movie 3

Supplementary Movie 4

Supplementary Movie 5

Supplementary Movie 6

Supplementary Movie 7

Supplementary Movie 8

Supplementary Movie 9
