# Supplementary material for: Injectable antibacterial conductive nanocomposite cryogels with rapid shape recovery for noncompressible hemorrhage and wound healing
Source: Nat Commun. 2018 Jul 17;9:2784. doi: 10.1038/s41467-018-04998-9 (PMC6050275; doi:10.1038/s41467-018-04998-9)
Supplement: Supplementary file 1 — Supplementary Information [file 41467_2018_4998_MOESM1_ESM.pdf]

Supplementary Information

Injectable Antibacterial Conductive Nanocomposite Cryogels with Rapid Shape Recovery for Noncompressible Hemorrhage and Wound Healing

Zhao et al.

Supplementary Figures

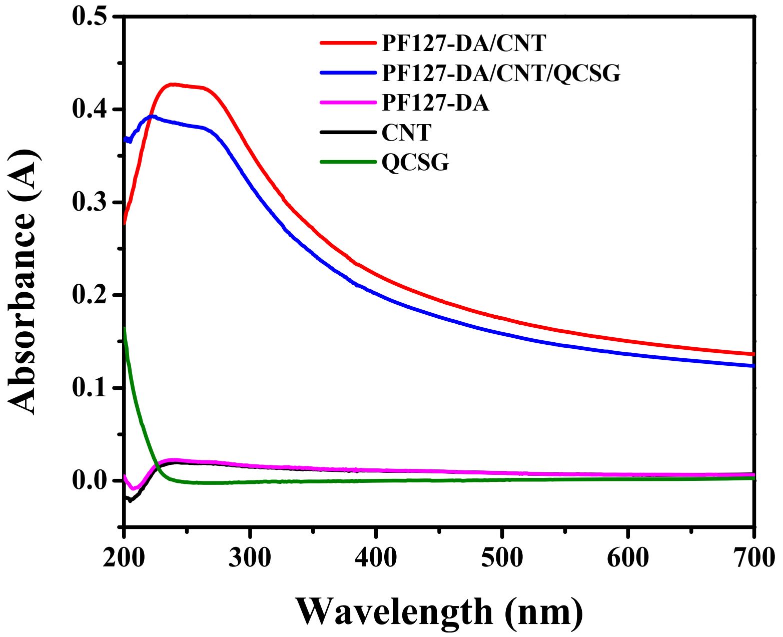

Supplementary Figure 1. UV-vis spectra of supernatants from CNT dispersion liquid, PF127-DA/CNT dispersion liquid, and PF127-DA/CNT/QCSG dispersion liquid, and solutions of PF127-DA and QCSG, respectively. The supernatant of pure CNT after ultrasound showed no characteristic absorption peaks for the strong van der Waals interactions in the CNT. After introducing PF127-DA, the supernatant of PF127-DA/CNT presented characteristic bands at 240 nm and 264 nm corresponding to absorption of 1D van Hove singularities[3]. This was because that the PF127-DA molecules were adsorbed on the surface of CNT via hydrophobic interaction and prevented re-aggregation resulting in stable PF127-DA/CNT dispersion (Supplementary Figure 2). The noncovalent hydrophobic interactions between polypropylene glycol (PPG) and CNT, hydrophilic PEO chain's extending in solution, and the strong physical interaction between QCSG and CNT all contributed to the stable dispersion of CNT in the matrix. Interestingly, in addition to the absorption peak at 264

nm, the UV-vis spectrum of supernatant from QCSG/PF127-DA/CNT showed a blue shift from 240 nm to 220 nm, suggesting the strong interaction between CNT and QCSG, which was probably cation-π interaction [4, 5, 6, 7, 8].

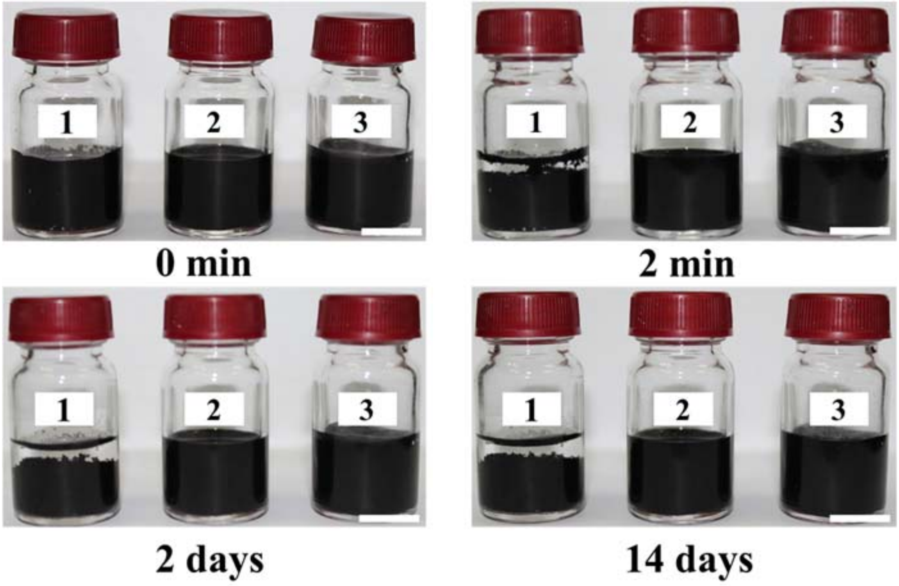

Supplementary Figure 2. Photographs of CNT dispersion liquid (1), PF127-DA/CNT dispersion liquid (2), and PF127-DA/CNT/QCSG dispersion liquid (3) after placed at room temperature for different time periods. Scale bar: 10 mm.

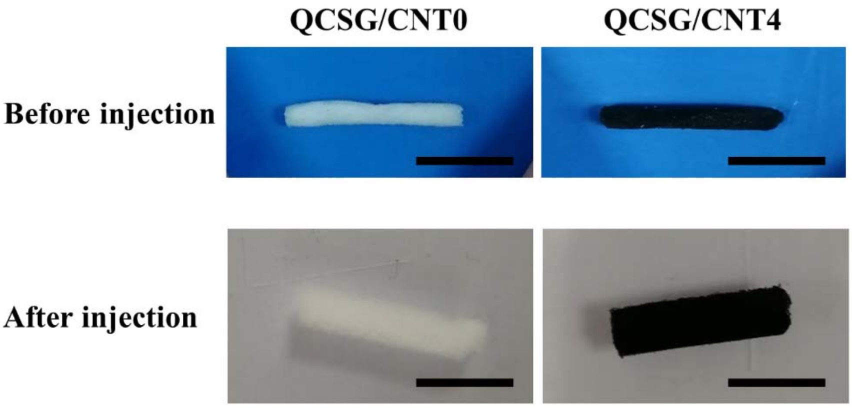

Supplementary Figure 3. Photographs of cryogel QCSG/CNT0 and cryogel QCSG/CNT4 before injection and after injection. Scale bar: 10 mm.

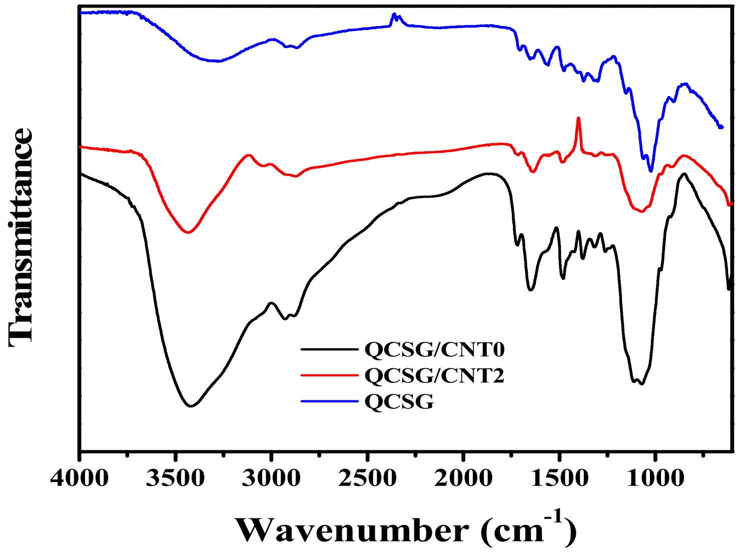

Supplementary Figure 4. FTIR spectra of QCSG/CNT0, QCSG/CNT2, and QCSG copolymer. QCSG presented two characteristic peaks at 1478 cm^{-1} and 1635 cm^{-1} assigned to the methyl bands of GTMAC [1] and double bonds of GMA[9], respectively. Compared with FTIR spectrum of QCSG, both QCSG/CNT0 and QCSG/CNT2 showed the characteristic peak of methyl bond of GTMAC at 1478 cm^{-1} and disappeared absorption peak of double bonds at 1635 cm^{-1}, revealing that the crosslinked cryogel networks of QCSG/CNT0 and QCSG/CNT2 were formed via free-radical polymerization of double bonds. All the above results demonstrated that the pure QCSG cryogel and QCSG/CNT cryogels were successfully synthesized by cryopolymerization under -20 °C with APS/TEMED as redox initiator system, and both PF127-DA and QCSG contributed to the good dispersion of CNT in the QCSG cryogel network leading to CNT-reinforced nanocomposite cryogels.

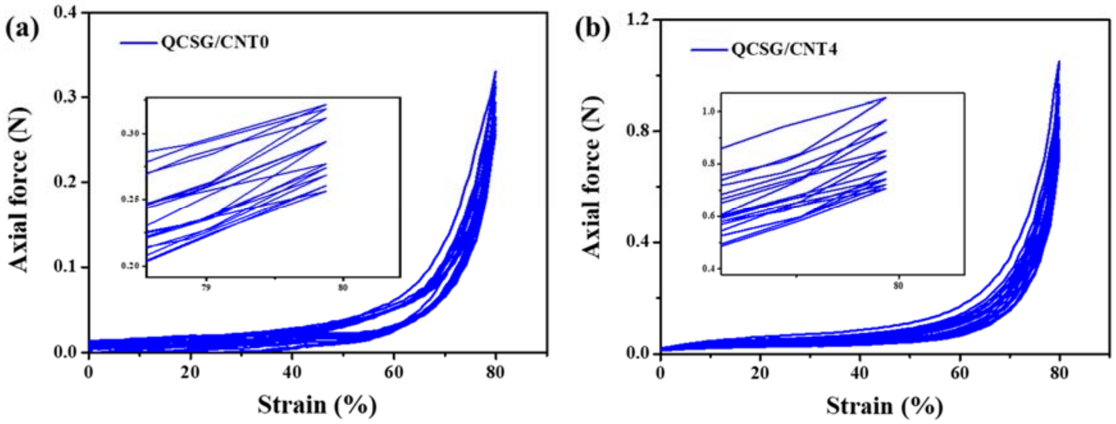

Supplementary Figure 5. Dynamic compression-strain curves of cryogel QCSG/CNT0 (a) and cryogel QCSG/CNT4 (b) at 80% strain for 100 cycles. When the cycles were further increased from 10 to 100 at 80% strain, QCSG/CNT0 and QCSG/CNT4 presented gradually increased recovery loss from 3.3% to 22.3% and from 11.8% to 32.9%, respectively (Supplementary Figure 5). However, all of the cryogels still kept good shape and elasticity, suggesting their great robustness.

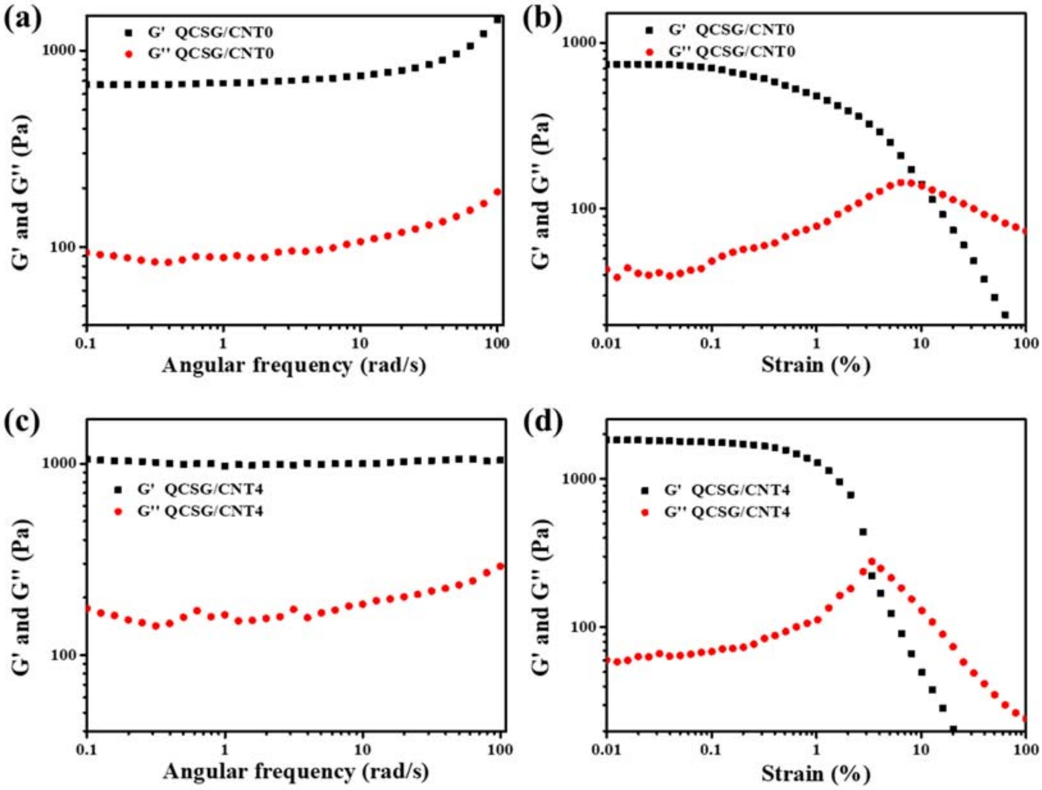

Supplementary Figure 6. Rheological properties of QCSG/CNT0 and QCSG/CNT4.

Both QCSG/CNT0 and QCSG/CNT4 showed stable or slightly increased G' and G'' when gradually increasing the frequency from 0.1 rad/s to 100 rad/s and fixing the strain at 1%, suggesting their stable cryogel networks. During the strain amplitude sweep test, both the cryogels showed gradually decreased G' and increased G'' when changing the strain from 0.01% to 100%, probably attributed to the rapid reversible shape deformation of the cryogel networks. QCSG/CNT4 presented higher storage moduli than those of QCSG/CNT0 due to the reinforcement of CNT.

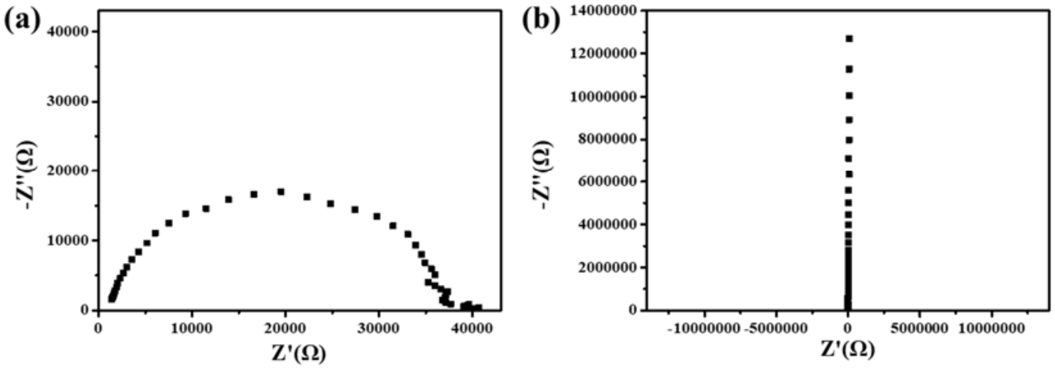

Supplementary Figure 7. Impedance curves of QCSG/CNT4 (a) and QCSG/CNT0 (b) with a frequency range of 10^2–10^6 Hz. The semicircle diameter of the cryogel QCSG/CNT4 at high frequency corresponding to electron-transfer resistance (R_{ct}) is about 36 kΩ (Supplementary Figure 7a), demonstrating that the cryogel QCSG/CNT4 is favorable for charge transfer. The R_{ct} of cryogel QCSG/CNT0 is almost infinite (Supplementary Figure 7b) for its formation of an insulating layer to inhibit electron transfer. Thus, the R_{ct} of cryogel QCSG/CNT4 dramatically decreases from infinite to 36 kΩ, implying that the incorporation of CNT endows cryogel with excellent electrical property.

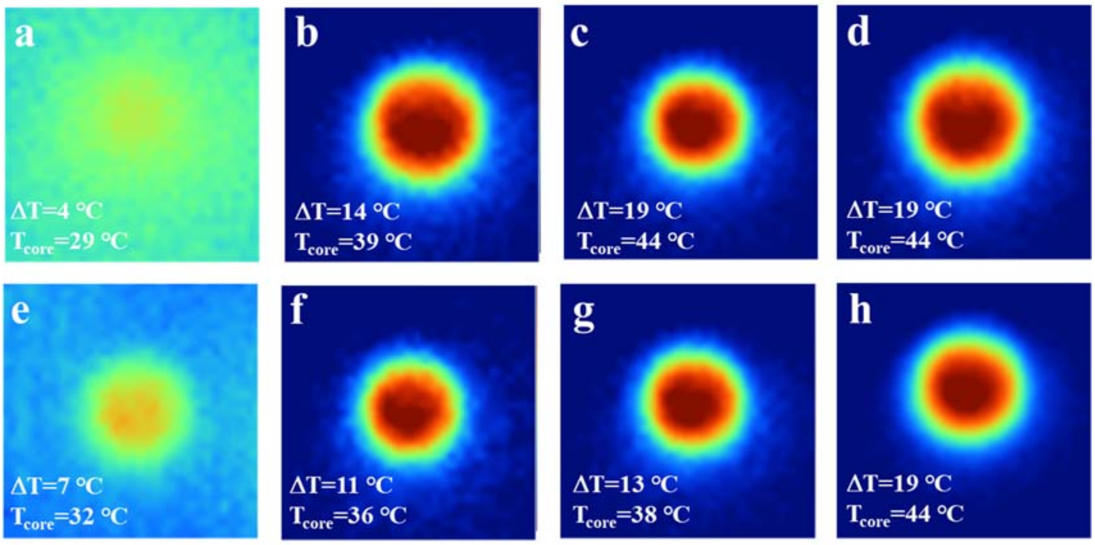

Supplementary Figure 8. Heat maps of the QCSG/CNT0 (a), QCSG/CNT2 (b), QCSG/CNT4 (c), and QCSG/CNT6 (d) after 10 min NIR 808 nm irradiation with a constant light intensity of 1.4 W/cm^2; Heat maps of QCSG/CNT4 after 10 min NIR 808 nm irradiation with the light intensity varying from 0.6 (e) to 0.9 (f), 1.1 (g) and 1.4 W/cm^2 (h), respectively.

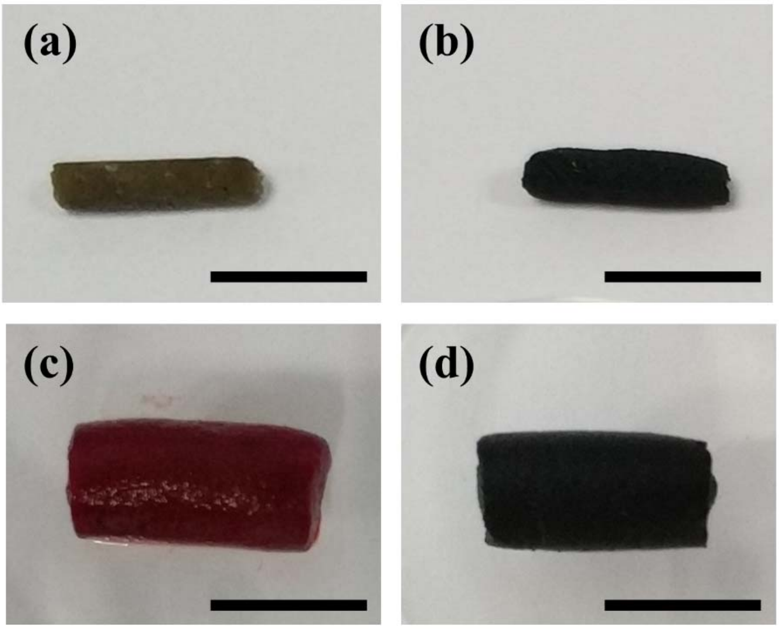

Supplementary Figure 9. Photographs of shape fixed QCSG/CNT0 (a) and QCSG/CNT4 (b); Photographs of QCSG/CNT0 (c) and QCSG/CNT4 (d) after

absorbing the blood. Scale bar: 10 mm.

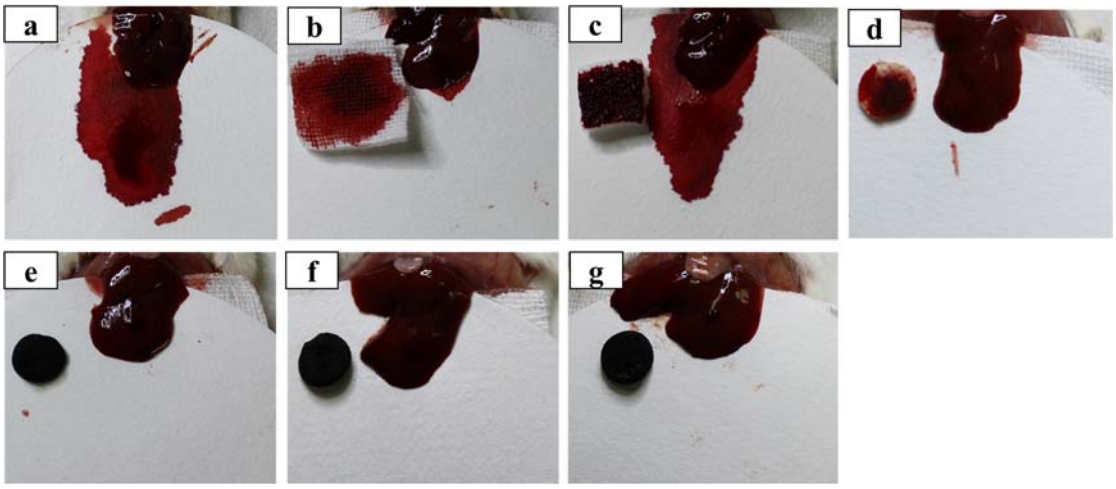

Supplementary Figure 10. Photographs of the hemostatic agent groups and blank group after *in vivo* hemostasis when used in a mouse liver injury model. (a): blank group; (b) gauze; (c) gelatin hemostatic; (d) QCSG/CNT0; (e) QCSG/CNT2; (f) QCSG/CNT4; and (g) QCSG/CNT6.

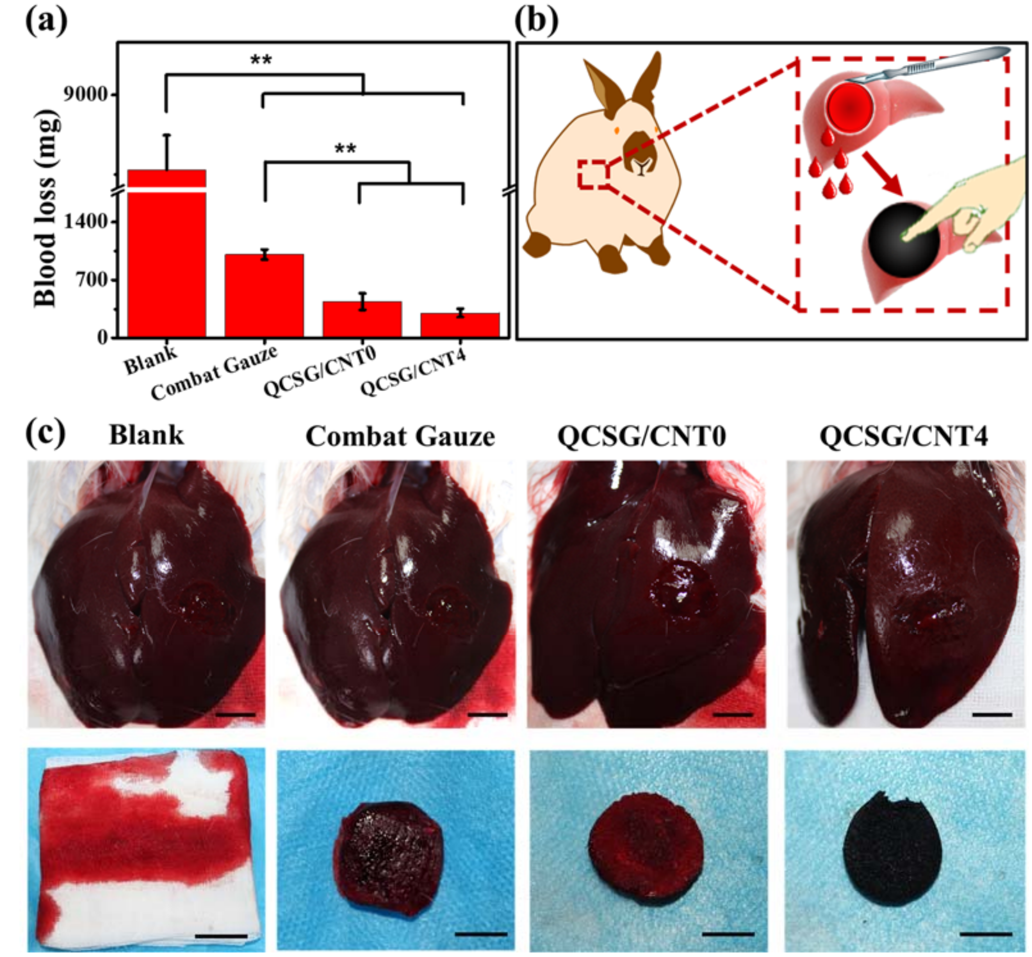

Supplementary Figure 11. (a) Hemostatic capacity evaluation of QCSG/CNT0, QCSG/CNT4 and Combat Gauze by using a standardized circular liver bleeding model; (b) Scheme representation of the standardized circular liver bleeding model during hemostasis; (c) Photographs of the hemostatic agent groups and blank group after *in vivo* hemostasis when used in a standardized circular liver bleeding model: blank group, Combat Gauze, QCSG/CNT0 and QCSG/CNT4. Scale bar: 8 mm. ** P<0.01 using Student t-test. Error bar indicates standard errors (n = 6).

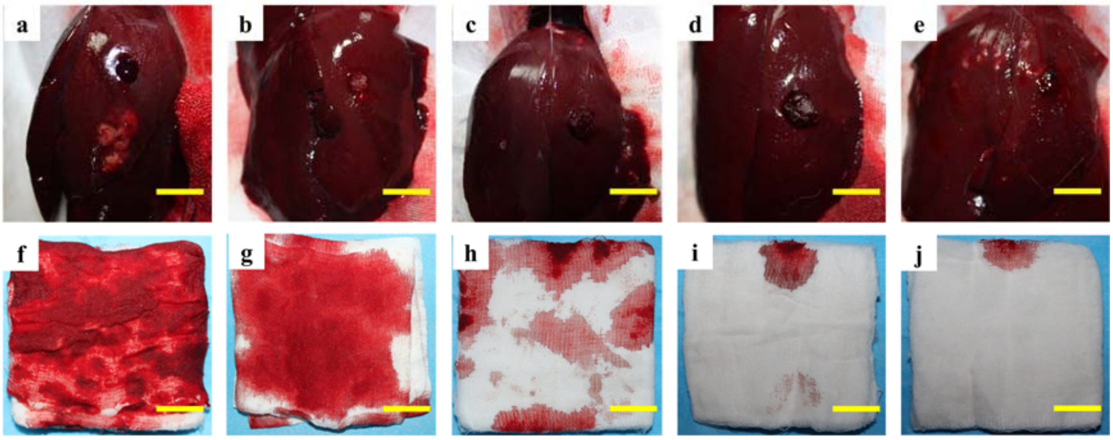

Supplementary Figure 12. Photographs of the hemostatic agent groups and blank group after *in vivo* hemostasis when used in rabbit liver volume defect lethal noncompressible hemorrhage model. Hemostatic agents inserted in the liver defect hole after hemostasis: (a) blank group; (b) gelatin hemostatic sponge D1; (c) gelatin hemostatic sponge D2; (d) QCSG/CNT0; and (e) QCSG/CNT4. Gauzes absorbing the bleeding blood during the hemostatic application: (f) blank group; (g) gelatin hemostatic sponge D1; (h) gelatin hemostatic sponge D2; (i) QCSG/CNT0; and (j) QCSG/CNT4. Scale bar: 10 mm.

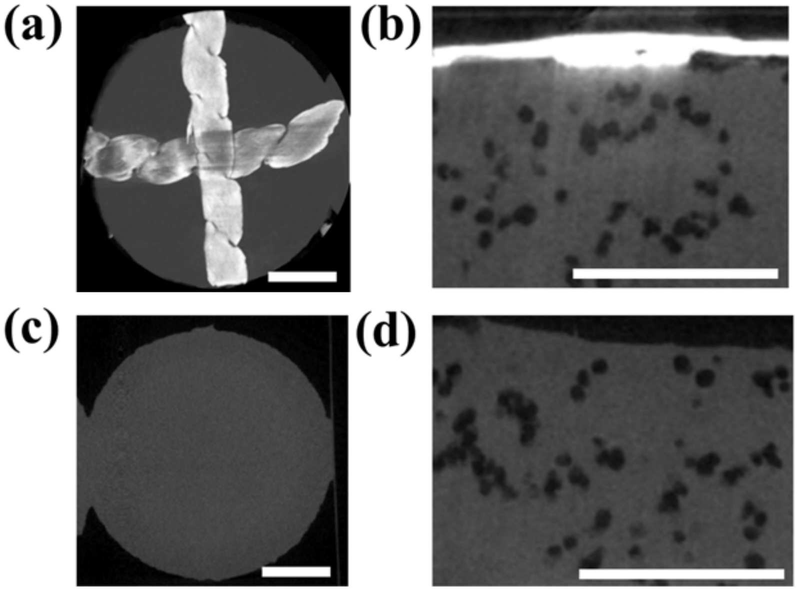

Supplementary Figure 13. The X-ray detectability was introduced to the cryogel by

gluing a X-ray detectable line to the cryogel, and the X-ray detectable line contained cryogels could be detected by Micro-CT clearly. (a) Top view of QCSG/CNT4 containing X-ray detectable line; (b) Longitudinal section view of QCSG/CNT4 containing X-ray detectable line; (c) Top view of QCSG/CNT4 without X-ray detectable line; (d) Longitudinal section view of QCSG/CNT4 without X-ray detectable line. Scale bar: 3 mm.

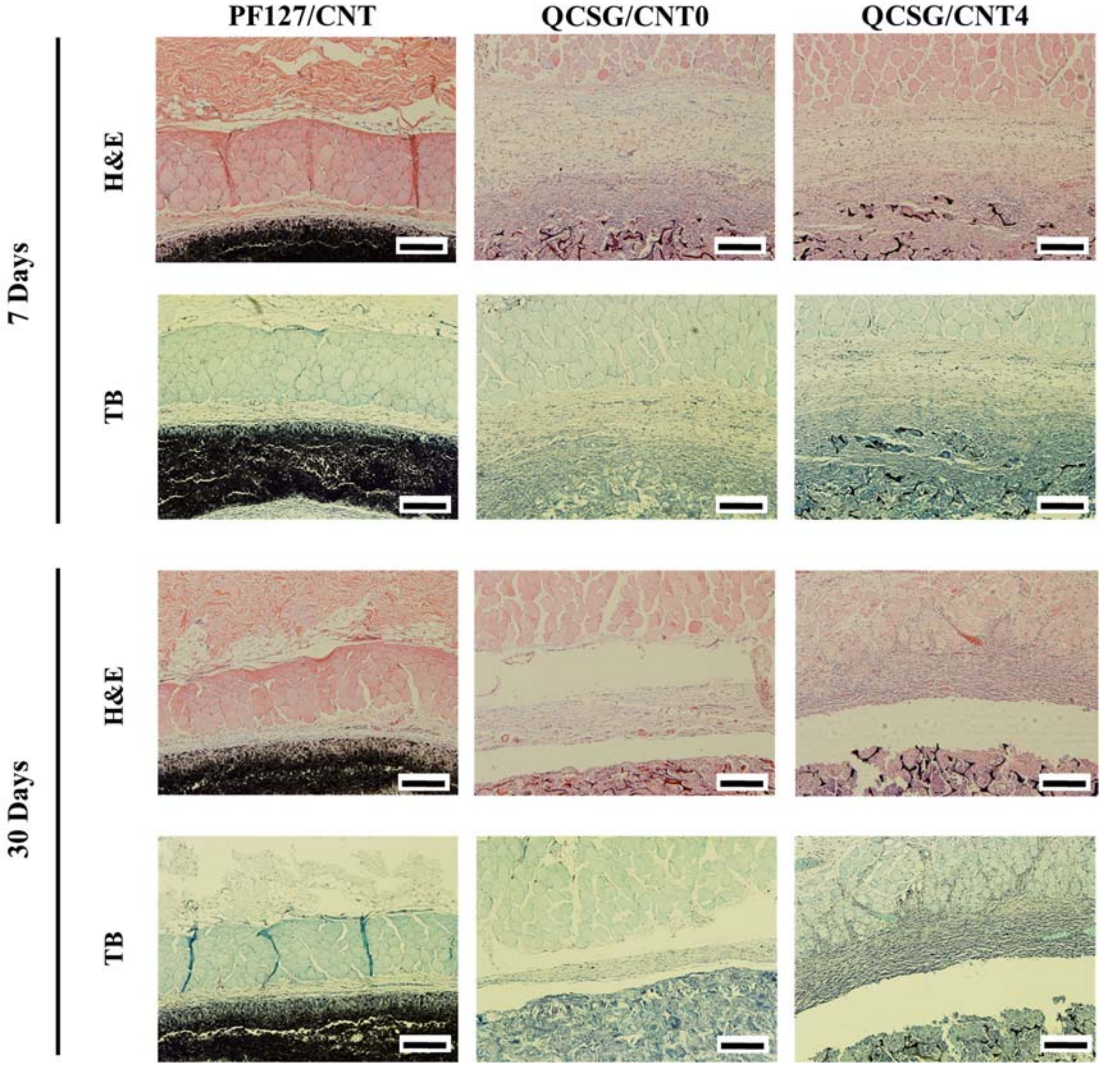

Supplementary Figure 14. H&E staining and toluidine blue staining results of PF127/CNT, QCSG/CNT0 and QCSG/CNT4 on day 7 and day 30. Scale bar: 200 μm.

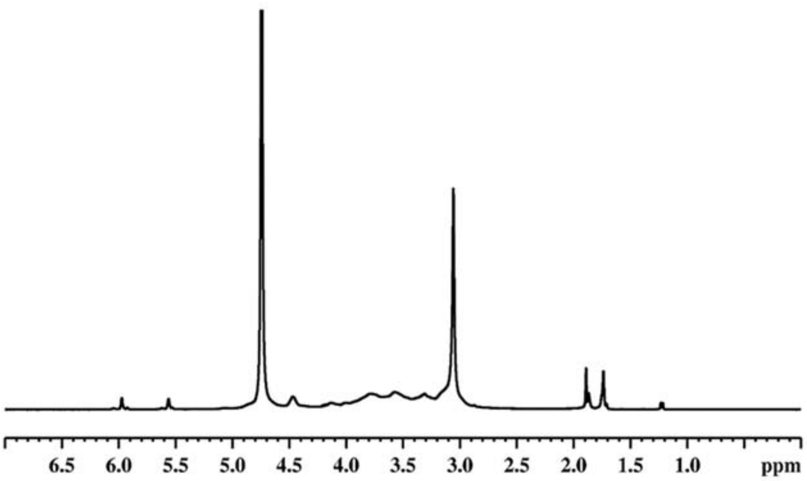

Supplementary Figure 15. ^{1}H NMR spectrum of QCSG. The characteristic peaks at 3.1 and 3.3 ppm corresponding to trimethylammonium groups and –NH–CH_2– groups [1] and two peaks at 5.6 ppm and 6.0 ppm assigned to double bonds from GMA were all observed in ^{1}H NMR of QCSG, suggesting the successfully synthesis of QCSG via one-pot reaction among chitosan, GTMAC and GMA.

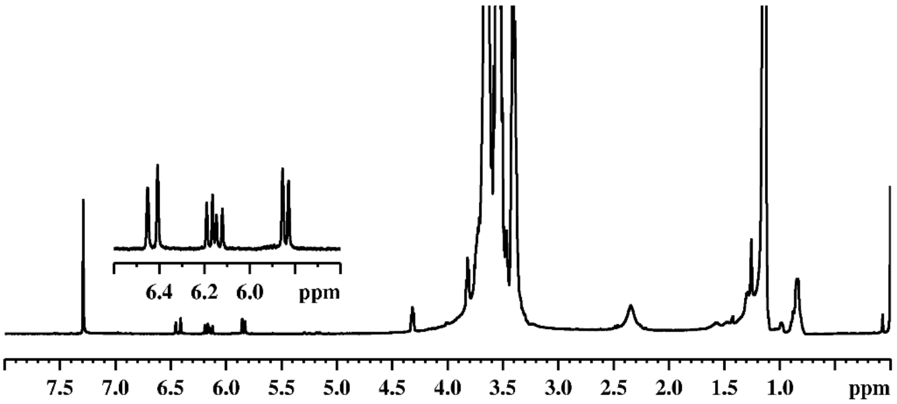

Supplementary Figure 16. ^{1}H NMR spectrum of PF127-DA. Two characteristic peaks at 5.8 and 6.4 ppm attributed to the double bonds from acrylate appearing in the spectrum of PF127-DA demonstrated the successful acylation of PF127 [10].

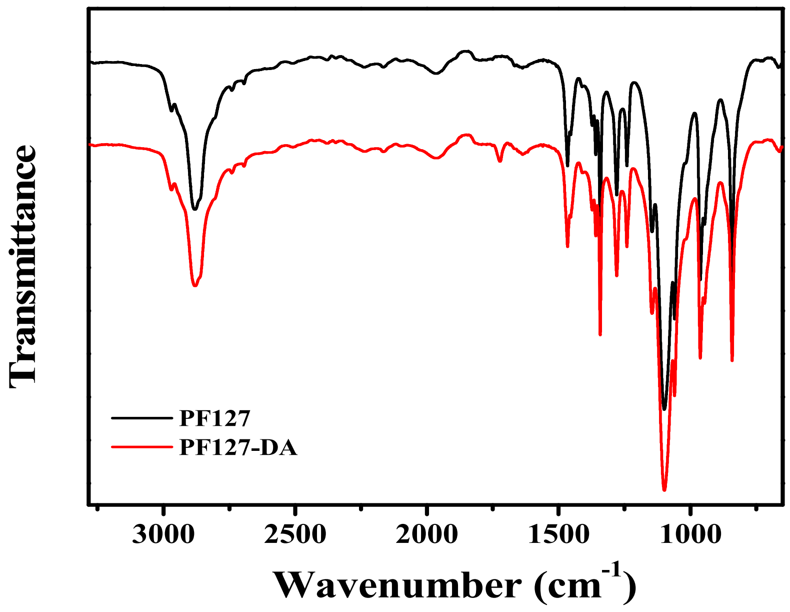

Supplementary Figure 17. FTIR spectra of PF127 and PF127-DA. Compared with the FT-IR spectrum of PF127, PF127-DA presented a new characteristic absorption peak of C=O stretching vibration at 1722 cm^{-1} from acryloyl chloride[10].

Supplementary Tables

Supplementary Table 1. The parameters of the synthesized QCSG copolymers.

Copolymer	Feed ratio of GTMAC to amino group	Feed ratio of GMA to amino group	Degree of GTMAC substitution	MIC	
				S. aureus (μg/mL)	*E. coli* (μg/mL)
QCSG1	1:1	0.5:1	23%	>2500	
QCSG2	2:1	0.5:1	35%	>2500	
QCSG3	3:1	0.5:1	46%	20	

Supplementary Table 2. The components of the cryogels. Four cryogels with a fixed 2.5 wt% of QCSG3 concentration and varying the CNT concentrations from 0 to 2, 4 and 6 mg/mL were synthesized. The four cryogels were coded as QCSG/CNT0, QCSG/CNT2, QCSG/CNT4 and QCSG/CNT6, and QCSG means QCSG3, while the number after CNT means the concentration (mg/mL) of CNT in the cryogel precursor.

Cryogel code	QCSG3 (mg)	PF127-DA (mg)	CNT (mg)	APS (mg)	TEMED (μL)
QCSG/CNT0	25	0	0	5	1
QCSG/CNT2	25	2	2	5	1
QCSG/CNT4	25	4	4	5	1
QCSG/CNT6	25	6	6	5	1

Supplementary Table 3. Volumetric expansion ratio and shape fixity ratio and recovery ratio of the cryogels. The cryogels with CNT concentrations of 0 mg/mL, 2 mg/mL and 4 mg/mL showed volumetric expansion ratios varying from 11 to 10.9 and 10.7. However, QCSG/CNT6 presented significantly decreased volumetric expansion ratio of 4.6. The fixity ratio results were consistent with the volumetric expansion ratios, in which all the cryogels showed relatively stable fixity ratios more than 88% except for QCSG/CNT6 just possessing fixity ratio less than 66% after five cycles.

Cryogel	V_r : V_f	R_{f1}, R_{r1} (%)	R_{f2}, R_{r2} (%)	R_{f3}, R_{r3} (%)	R_{f4}, R_{r4} (%)	R_{f5}, R_{r5} (%)
QCSG/CNT0	11.0	89.4, 100	89.4, 100	89.4, 100	88.8, 100	88.2, 100
QCSG/CNT2	10.9	88.0, 100	90.5, 100	92.8, 100	92.8, 100	92.0, 100
QCSG/CNT4	10.7	89.3, 100	89.3, 100	89.3, 100	88.7, 100	88.7, 100
QCSG/CNT6	4.6	62.7, 100	62.7, 100	63.9, 100	64.7, 100	66.4, 100

V_r represents recovery cryogel volume while V_f represents shape fixed cryogel volume.
R_f represents the cryogel fixity ratio recovery while R_r represents cryogel shape recovery ratio.
The number in R_f and R_r means the repeat times during shape memory test.

Supplementary Notes

Supplementary Note 1. CNT stability of the cryogels

When injecting or implanting the cryogel in bleeding site or wound, the cryogel would bear continuous and dynamic force loading. In order to evaluate the CNT stability of the cryogel when bearing continuous and dynamic compression, a dynamic compression test (50% strain) was performed as long as 6 h (100 cycles) with QCSG/CNT4 soaked in DI water, and QCSG/CNT4 remained more than 99.2% CNT within its matrix after suffering the dynamic compression test. The tested QCSG/CNT4 also maintained about 99.0% CNT when immersed in DI water at 37 °C with a shaking speed of 100 rpm for another 24 h. Furthermore, the QCSG/CNT4, after suffering a rather extreme dynamic compression test of 100 cycles at 80% strain, also showed more than 98.1% of CNT stably entrapped in the cryogel network when further immersed in DI water for 24 h, attributed to the physical interactions between CNT and PF127-DA. When delivering the cryogel to bleeding site, the cryogel rapidly absorbed blood and accelerate blood coagulation during hemostatic application, and the coagulated blood further strengthened the cryogel network and crosslinked with CNT to prevent the CNT release from the cryogel.

Supplementary Note 2. Cryogels remaining safe compression moduli after application

During the hemostatic application, the shape-fixed cryogels can absorb blood and then recover their shapes. The cryogel will remain a certain compression strain due to the

limited space from surrounding tissues. However, with the increase of CNT from 0 to 6 mg/mL, the cryogels just presented about 0.49 kPa to 1.19 kPa compression moduli when remaining 20% strain. These compression moduli are lower than those of human soft tissues (between several kPa to several MPa) [11, 12, 13, 14]. Therefore, the cryogel hemostatic agents will not cause severe pressure and additional injury to soft tissue during the application.

Supplementary Note 3. Mechanism of the high resilience and rapid recovery behavior of the cryogels

In the first stage of compression, the macroporous sponge-like cryogel matrix could allow water freely flow in and out, and just experienced collapsed macropores and deformed matrix under the compressive stress. The water would be squeezed out from the cryogel and then effectively relieve the internal compressive stress of cryogel, which just led to little strain deformation. Once the stress was removed, the cryogel would instantly recover and absorb the water back simultaneously, which was derived by the bended cryogel network. When further increasing the compression stress to a high level, the cryogel matrix would relieve most of the compression stress by squeezing out of the water, and then the network of CNT hard microdomain physical crosslinker would serve as a highly resilient substrate to prevent the mechanical failure of the cryogel [2, 15].

Supplementary Note 4. Photothermal antibacterial activity of the cryogels

As shown in Figure 3e-j, when not applied NIR irradiation, PBS groups presented no bacteria log reduction for *S. aureus*, *E. coli* and *P. aeruginosa*, while QCSG/CNT0 and QCSG/CNT4 showed log reductions of 0.16 and 0.17 for *S. aureus*, 0.23 and 0.20 for *E. coli*, and 0.23 and 0.25 for *P. aeruginosa*, respectively, due to QCSG's inherent antibacterial activity. After applying NIR irradiation (1.4 W/cm^2) for 20 min, the PBS groups showed weak bacteria log reductions from 0.07 to 0.13 for *S. aureus*, from 0.02 to 0.04 for *E. coli*, and from 0.01 to 0.14 for *P. aeruginosa*, respectively, while QCSG/CNT0 group showed slightly enhanced bacteria log reductions from 0.16 to 0.36 for *S. aureus*, unchanged bacteria log reductions about 0.23 for *E. coli*, and slightly enhanced bacteria log reductions from 0.23 to 0.36 for *P. aeruginosa*, respectively. This was because PBS and QCSG/CNT0 presented negligible photo-thermal capability (Figure 3b), and the obviously enhanced *S. aureus* killing ratio after NIR irradiation might be due to higher sensitivity of *S. aureus* to NIR irradiation than those of *E. coli* and *P. aeruginosa*.

Supplementary Note 5. Cytocompatibility evaluation of the cryogels

After contacting with the cryogel surface for 24 h, all the cells in the three CNT-contained cryogel groups showed more than 90% cell viability compared with the TCP control group ($P>0.05$) (Figure 5d). However, QCSG/CNT0 group showed the lowest cell viability of 75%, which was significantly lower than CNT-contained cryogel groups and TCP group ($P<0.05$). Consistent with the results in Figure 5d, most of cells in all

the four cryogel groups were green and showed spindle-like shape similar to that of TCP control group. Few dead cells in all the test groups were caused by cell metabolism and apoptosis. However, QCSG/CNT0 group showed obviously reduced cell number compared to CNT-contained groups and TCP group, suggesting slight inhibition of cell proliferation of QCSG/CNT0 due to its strong positive-charged nature [1]. Interestingly, the incorporation of appropriate content of CNT into cryogel network increased cytocompatibility of QCSG by the cation-π interaction between QCSG and CNT [16].

Supplementary Note 6. *In vitro* blood clotting performance of the gauze and gelatin hemostatic sponge

A few blood cells were observed in gauze group while slightly enhanced number of blood cells was presented in gelatin hemostatic sponge group, and the blood cells in both the two groups kept their distinctive biconcave disks. Besides, gauze group almost showed no platelet adhesion, while gelatin hemostatic sponge presented few platelets. Although gelatin hemostatic sponge group exhibited enhanced blood cell adhesion and platelet activation, but it showed less efficient blood clotting than gauze group (Figure 6a), which is because gauze could rapidly absorb blood to concentrate the blood to induce blood clotting, while the gelatin hemostatic sponge couldn't rapidly absorb blood into its network and just provide a hemostatic surface to induce blood clotting.

Supplementary Note 7. *In vivo* hemostasis for lethal noncompressible hemorrhage

QCSG/CNT4 possessing excellent *in vivo* hemostatic capability compared to

QCSG/CNT0 was chosen to perform the lethal noncompressible hemorrhage hemostasis. QCSG/CNT0, gelatin hemostatic sponge D1 (with a diameter of 4 mm) and gelatin hemostatic sponge D2 (with a diameter of 6 mm) were used as control groups. As shown in Figure 7g-i, the rabbits in blank group presented the most blood loss of 24.5 g and the longest blood bleeding time of 25.0 min, and all the rabbits were dead within 25 min. These results demonstrated that the liver volume defect model is a lethal noncompressible hemorrhage model, in which the animal loses a large amount blood within short time and the rabbit cannot stop the bleeding by its own hemostatic capacity.

Supplementary Note 8. *In vivo* hemostasis of gelatin hemostatic sponges for lethal noncompressible hemorrhage

Although the gelatin hemostatic sponge D1 possessed the same diameter with the shape fixed cryogel groups, it had no shape recovery property after contacting with blood. Therefore, gelatin hemostatic sponge D1 with permanent diameter slightly smaller than wound could just partly block the liver defect hole, causing significantly reduced blood loss than blank group ($P<0.001$) (Figure 7g). Although, gelatin hemostatic sponge D2 with a diameter bigger than wound presented better hemostatic effect than gelatin hemostatic sponge D1 due to its capacity to completely block wound except for its inherent hemostatic ability, it presented weak mechanical strength (especially in the state after absorbing blood) limiting its hemostatic efficiency on lethal noncompressible hemorrhage application. Furthermore, the use of gelatin sponge was not convenient when the bleeding was noncompressible and the wound was narrow, deep and irregular.

Supplementary Note 9. Discussions on the safety of cryogels for hemostasis

For clinic application, to stop the deep and narrow wound bleeding for human body, the cryogels with greater height (such as more than 4 cm) can be prepared to ensure that part of the cryogel is exposed outside after injection, and the surgeon can inject columnar cryogel hemostatic agent with proper size into the bleeding site. In addition, for massive hemorrhage on the body surface (such as leg amputation), the surgeon can use disk shape cryogel with proper surface area according to the size of the specific wound, thus the material will not enter the body for its much bigger diameter than that of the blood vessel (The blood vessel diameters of the legs are no more than 14 mm)[17].

Supplementary Note 10. *In vivo* wound healing performance of the cryogels

Although there is some limitation about using mouse skin as wound healing model [18], mice were chosen for wound-healing study for several reasons: availability, low cost, the ability to test large numbers of animals with reproducible results, and the potential to test a variety of genetic knockout animals [19]. Both the wound contraction and histomorphological evaluation results on 5th, 10th and 15th day were used to evaluate the healing effect of the cryogels with commercial film dressing (Tegaderm™) as a control group.

Supplementary Note 11. *In vivo* host response of the cryogels

The *in vivo* subcutaneous transplantation of QCSG/CNT0, QCSG/CNT4 and PF127/CNT dispersion for 7 days and 30 days was performed to evaluate their acute

and chronic inflammatory responses. As shown in Supplementary Figure 14, the H&E staining results revealed that all of the three samples presented mild acute inflammatory responses after implantation for 7 days. The PF127/CNT dispersion liquid just showed thin fibrous inflammatory zone, and both the QCSG/CNT0 and QCSG/CNT4 also showed similar thickness of fibrous inflammatory zones. Toluidine blue staining results at day 7 also indicated the very mild acute inflammatory responses of the three samples, and they showed several and similar number mast cells around the materials. After implantation for 30 days, the H&E staining results showed that all of the three samples exhibited quite dense fibrous inflammatory zones. The toluidine blue staining results on day 30 also demonstrated that the number of mast cells obviously decreased in all of the three samples. The results proved the very mild inflammatory responses of the three samples. Thus, these cryogels could be used as biocompatible temporary cryogel hemostatic agents for *in vivo* application.

Supplementary References

1. Zhao X, Li P, Guo B, Ma PX. Antibacterial and conductive injectable hydrogels based on quaternized chitosan-graft-polyaniline/oxidized dextran for tissue engineering. *Acta biomaterialia* 2015, **26:** 236-248.
2. Liu Y, Xu K, Chang Q, Darabi MA, Lin B, Zhong W, *et al.* Highly Flexible and Resilient Elastin Hybrid Cryogels with Shape Memory, Injectability, Conductivity, and Magnetic Responsive Properties. *Advanced Materials* 2016, **28**(35)**:** 7758-7767.
3. Yu J, Grossiord N, Koning CE, Loos J. Controlling the dispersion of multi-wall carbon nanotubes in aqueous surfactant solution. *Carbon* 2007, **45**(3)**:** 618-623.
4. Shang S, Gan L, Yuen MC-w. Improvement of carbon nanotubes dispersion by chitosan salt and its application in silicone rubber. *Compos Sci Technol* 2013, **86:** 129-134.
5. Yamada S, Misono T, Tsuzuki S. Cation− π interactions of a thiocarbonyl group and a carbonyl group with a pyridinium nucleus. *J Am Chem Soc* 2004, **126**(31)**:** 9862-9872.
6. Yan LY, Poon YF, Chan-Park M, Chen Y, Zhang Q. Individually dispersing single-walled carbon nanotubes with novel neutral pH water-soluble chitosan derivatives. *J Phys Chem C* 2008, **112**(20)**:** 7579-7587.
7. Meyer F, Minoia A, Raquez JM, Spasova M, Lazzaroni R, Dubois P. Poly (amino-methacrylate) as versatile agent for carbon nanotube dispersion: an experimental, theoretical and application study. *J Mater Chem* 2010, **20**(33)**:**

6873-6880.

8. Nivethaa E, Dhanavel S, Narayanan V, Stephen A. Fabrication of chitosan/MWCNT nanocomposite as a carrier for 5-fluorouracil and a study of the cytotoxicity of 5-fluorouracil encapsulated nanocomposite towards MCF-7. *Polym Bull* 2016, **73**(11)**:** 3221-3236.

9. Zhao J, Zhao X, Guo B, Ma PX. Multifunctional interpenetrating polymer network hydrogels based on methacrylated alginate for the delivery of small molecule drugs and sustained release of protein. *Biomacromolecules* 2014, **15**(9)**:** 3246-3252.

10. Niu G, Zhang H, Song L, Cui X, Cao H, Zheng Y*, et al.* Thiol/acrylate-modified PEO-PPO-PEO triblocks used as reactive and thermosensitive copolymers. *Biomacromolecules* 2008, **9**(10)**:** 2621-2628.

11. Serrano MC, Chung EJ, Ameer GA. Advances and Applications of Biodegradable Elastomers in Regenerative Medicine. *Adv Funct Mater* 2010, **20**(2)**:** 192–208.

12. You Z, Wang Y. *Bioelastomers in Tissue Engineering*. Springer Vienna, 2011.

13. Meyers MA, Chen PY, Lin YM, Seki Y. Biological materials: Structure and mechanical properties. *Prog Mater Sci* 2008, **53**(1)**:** 1-206.

14. Fung YC, Skalak R. *Biomechanics:Mechanical properties of living tissues*. Springer-Verlag, 1981.

15. Annabi N, Shin SR, Tamayol A, Miscuglio M, Bakooshli MA, Assmann A*, et al.* Highly elastic and conductive human-based protein hybrid hydrogels. *Adv*

Mater 2016, **28**(1)**:** 40-49.

16. Shi L, Zhang W, Yang K, Shi H, Li D, Liu J, *et al.* Antibacterial and osteoinductive capability of orthopedic materials via cation–π interaction mediated positive charge. *J Mater Chem B* 2015, **3**(5)**:** 733-737.

17. Pang Gang, Long ZW. *Clinical anatomy of human blood vessel and vascular anastomosis*. People's Medical Publishing House, 2010.

18. Jung Y, Son D, Kwon S, Kim J, Han K. Experimental pig model of clinically relevant wound healing delay by intrinsic factors. *International Wound Journal* 2013, **10**(3)**:** 295-305.

19. Wu JC, Rose LF, Christy RJ, Kai PL, Chan RK. Full-Thickness Thermal Injury Delays Wound Closure in a Murine Model. *Advances in Wound Care* 2015, **4**(2)**:** 83.
