# Supplementary material for: Injectable antibacterial conductive nanocomposite cryogels with rapid shape recovery for noncompressible hemorrhage and wound healing
Source: Nat Commun. 2018 Jul 17;9:2784. doi: 10.1038/s41467-018-04998-9 (PMC6050275; doi:10.1038/s41467-018-04998-9)
Supplement: Supplementary file 2 — Description of Additional Supplementary Files [file 41467_2018_4998_MOESM2_ESM.pdf]

Description of Additional Supplementary Files

File Name: Supplementary Movie 1
Description: Injection process of QCSG/CNT0 cryogel with free shape diameter of 8 mm via an injector with diameter of 4 mm.

File Name: Supplementary Movie 2
Description: Injection process of QCSG/CNT4 cryogel with free shape diameter of 8 mm via an injector with diameter of 4 mm.

File Name: Supplementary Movie 3
Description: Injection process of QCSG/CNT0 cryogel with free shape diameter of 5 mm via an injector with inner diameter of 1.5 mm.

File Name: Supplementary Movie 4
Description: Injection process of QCSG/CNT4 cryogel with free shape diameter of 5 mm via an injector with inner diameter of 1.5 mm.

File Name: Supplementary Movie 5
Description: Dynamic compression test of cryogel QCSG/CNT0 using rheometer.

File Name: Supplementary Movie 6
Description: Dynamic compression test of cryogel QCSG/CNT4 using rheometer.

File Name: Supplementary Movie 7
Description: Shape recovery of the cryogels after absorbing the water.

File Name: Supplementary Movie 8
Description: Blood absorbing process of cryogel QCSG/CNT4.

File Name: Supplementary Movie 9
Description: Blood absorbing process of cryogel QCSG/CNT0.
